# A Dynamic Thermal-Mechanical Coupling Numerical Model to Solve the Deformation and Thermal Diffusion of Plates

**DOI:** 10.3390/mi13050753

**Published:** 2022-05-10

**Authors:** Wenxing Chen, Shuyang Dai, Baojuan Zheng

**Affiliations:** 1 School of Mathematics and Statistics, Wuhan University, Wuhan 430072, China; wenxingchen@whu.edu.cn; 2 Hubei Key Laboratory of Computational Science, Wuhan University, Wuhan 430072, China; 3 Commerical Vehicle Technology Center, SAIC Motor Group Co., Ltd., Shanghai 200041, China; zhengbaojuan2020@163.com

**Keywords:** multi-physics model, bending and deformation, crack growth, graphene, laser heating, monocrystalline silicon plate

## Abstract

Elastic materials include metal plates, rubber, foam, airbags and so on, which have a good buffer effect, toughness and strong recovery ability. In this paper, the deformation and thermal diffusion of 2D and 3D thin plates are studied. Two models are established for the deformation of 2D thin plates. The bending deformation equation of rectangular and circular plates is derived, and the semi-analytical solution of the deflection function w(x,y) is found through the Fourier series approximation in the polar coordinate. The consistencies of the numerical solution and the theoretical solution are verified by numerical method. Then, we find that the factors affecting the deformation are related to the Young’s modulus, load, plate length and deformation factor α of the material. In a separate temperature physics field, we establish a heat conduction model of 2D graphene film. Three numerical schemes of the transient heat conduction equation of FDM-FEM are given. In contrast, this paper uses the implicit Euler method to discrete the time term. Furthermore, we compared the difference between the adiabatic condition and the convection condition by the graphical method and the curve trend. The results show that the temperature near the adiabatic boundary is higher. Finally, we proposed a 3D dynamic thermal–mechanical coupling model (3D-DTMCM) that has been established. A laser heating monocrystalline silicon sheet with periodic motion formula is given. The temperature radiation of the laser heat source has Gaussian distribution characteristics. Our proposed model can dynamically determine Young’s modulus with a variable temperature. The numerical results show that the higher the temperature is, the higher the strain energy density of the plate is. In addition, the deformation amplitude of the plates in the coupling field is larger than that in the single mechanical field. Finally, we also discussed the stress field distribution of mixed cracks under high temperature and high load. Our research provides theoretical support for the deformation of different plates, and also reflects the value of the coupled model in practical applications.

## 1. Introduction

### 1.1. Research Motivation and Significance

Nowadays, more and more new materials have entered the field of vision of scientists. The study of deformable materials has become a hot topic in this field, and it is also very important in the design and application of materials. The mechanical changes such as deformation, tension, torsion and fracture of materials are the inherent mechanical properties of objects. The application of deformed materials in real life and industry is also relatively common. The biggest problems facing the deformation of materials are the accurate description of deformation variables and the physical deformation modeling containing temperature or material with crack defects. In addition, the deformation adaptability of different materials is low when the material has defects. Many scientific experiments show that temperature and deformation are closely related, and a multiphysics theoretical model of mechanical and thermal coupling is expected to be established. However, the theory of studying deformation problems in multiphysics models is still in the exploratory stage. Therefore, the current status also reflects the research value of our paper and the problems that need to be solved urgently. In this paper, the coupled physical model is used to solve the material deformation. The purpose of the research is to improve the accuracy of the model and expand the application range, so that the model we established can solve the deformation of the thin plate material in the high temperature environment. Our method provides a better theoretical basis for mechanical performance changes of plates in the thermo-mechanical coupled Fields and single fields.

### 1.2. Related Work

Recently, the 3D printing technology [1], robot software organization [2,3], self-healing display screen [4], wearable electronic materials and battery expansion deformation are promoted [5,6]. These materials have a common deformation of porous materials deformation, followed by deformation related to temperature [7,8,9,10]. The theory and model of these problems are still in the exploratory stage, and there are still many areas to be improved in traditional numerical methods. In recent years, there have been many numerical methods for studying the deformation of materials, mainly based on deformation and other physical models to solve various practical problems. Valerio et al. used the virtual displacement principle and Ritz method to solve the problem of circular plate radial bending, which needs to derive the specific approximation function of the displacement field [11]. The ZOP model is used to solve the prediction of the thermal deformation of metal materials. The modified ZOP model can be used to predict the yield stress and reflect the peak stress [12]. This model can effectively reflect the occurrence and completion of dynamic recrystallization in the thermal deformation of metal materials. Titanium aluminum materials are prone to shear deformation at high temperatures. High temperature annealing at *t* = 1000 °C for 0.5–4 h also affects the structure and phase composition of the material [13]. The superconducting properties and technology of a high temperature superconductor (HTS) are constantly improving, and the shape variables of the conductor at ultra-low temperature and high temperature are also different.

Temperature affects material deformation in many cases: at 250–450 °C, Reference [14] used the improved hyperbolic sine method to calculate the activation energy and stress index. It was found that the plasticity of RE (AZ31—1RE) alloy after heating was enhanced, and the optimal strain rate and temperature range of hot processing were also determined [15]. With the development of composite materials, it also included the influence of Ca and Sc addition in rare earth materials and non-rare earth materials on the thermal deformation behavior, recrystallization and texture evolution of alloy materials [16]. The mechanical behavior and microstructure evolution of the plastic deformation of TC4 titanium alloy also occur at high temperatures of 900–1100 °C. The results show that TC4 titanium alloy has an obvious discontinuous yield phenomenon under high temperature deformation conditions, and the higher the deformation temperature is, the more obvious the discontinuous yield phenomenon is. At a low strain rate, TC4 titanium alloy first shows slight strain hardening in a small strain range, and then shows the characteristics of continuous softening flow, and finally tends to be stable. Reference [17] established the linear and nonlinear changes of thermal stress of FGM plate under thermal load, and the in-plane temperature changed in accordance with sine.

There are many ways to study the influence of temperature on material deformation. One of them is the multi-physical field of mechanical-thermal coupling by FEM method to solve material deformation [18,19,20]. For example, the thermal coupling elastic large deformation model of multi-layer functionally graded material curved beams with arbitrary undeformed configuration is established, which solves the difficulty of determining the neutral axis position of the beam in the classical analysis. The numerical results show that the graded material curved beams will have various jump phenomena under thermal-mechanical load [21]. Coupling analysis can solve relatively complex physical problems. The calculation is also large. The electrical-thermal-stress (ETS) modeling of semiconductor devices requires the use of finite element analysis (FEA) for device simulation. The static characteristics and recovery transient characteristics of the power diode are also considered. The S-E model of the power diode is calculated by using the variable situation theory [22]. In addition, the most popular machine learning has also been introduced into the industrial and academic fields. The appropriate neural network is used, and then the data-driven material constitutive model is combined with the finite element to realize the accurate description of the mechanical behavior of lithium metal under different temperatures and deformation scenarios. Finally, the temperature-stress-rate-deformation behavior of lithium metal can be predicted [23]. The biggest problem of this method is that the internal parameter transmission law is not completely clear, and the adjustment of model parameters and the labeling of training data need experience.

Thin plate bending is the most common physical phenomenon in engineering and life, which belongs to the knowledge of elastic mechanics [24,25]. When the special regular shape and the specific boundary conditions are satisfied, the deformation problem generally has an analytical solution. For example, the rectangular plate with four edges fixed can be solved by the superposition method, the deformation of the circular plate can be solved by axisymmetric deformation method, and the stress problem of the elliptical plate along the symmetric axis [26]. However, it is very difficult to find an analytical solution for the loading of the variable force on the boundary of the asymmetric graph, which can only be solved by a numerical method. In addition, the traditional numerical method is relatively small in solving the elastic deformation with temperature effect [27,28]. The coupling of multiple physical fields is a solution idea, but with an additional equation group, the solution time will increase, and the coupling and iterative convergence solution will be high. In this paper, another elastic deformation model based on the energy method is given in the sense of constant temperature and identity.

In addition, the bending of the plate is related to material properties, external load and ambient temperature. On the analysis of the influence of thermal load on the dynamic bending response of the clamped plate, it can also be considered (WBM). The wave-based model of the dynamic plate bending problem under thermal load is established, and its convergence rate is higher than that of FEM [29]. Thermal load also has important applications in crack problems. Reference [30] also discussed the adiabatic condition and vanishing temperature condition. They used the complex potential method to analyze the temperature field and stress field and also explained the difference between the two temperature conditions. The two-scale analysis framework with modified SCA method can be efficient in simulating composites considering thermal residual strains and stresses [31]. However, the establishment of thermal stress analysis cases under multi-physical fields is relatively more universal. For plate problems, there is a fixed solution model that is conducive to industrialization. Based on computational fluid dynamics (CFD), the subsequent heat of the whole panel and the thermal deformation and thermal stress caused by neutral particles are analyzed [32]. In addition, an efficient evaluation method for automobile shells design based on machine learning, this also belongs to the study of material deformation in multi-physical fields [33]. There is a lot of software to solve thermal stress and thermal deformation, such as COMSOL, ANSYS, ABAQUS, ADINA, etc. [34,35,36,37,38,39]. At the same time, we hope that more researchers can expand and innovate more numerical methods of coupled model.

The single-layer single deformation research in this paper also provides a good foundation for the deformation research of composite laminates. The deformation of laminates with cracks is a very challenging subject. There is sufficient experimental evidence that the propagation of cracks in brittle, isotropic and homogeneous materials makes the crack tip maintain the pure type I condition. The competition between the crack propagation in the interface and the bending out of the interface depends on the relative toughness of the material. For homogeneous isotropic solids, the role of the interfacial stress intensity factor is exactly the same as that in elastic fracture mechanics [40]. We need to refer to some classical models when studying the bending and deformation of some plate materials or crystal materials, and then modify and improve some theories, especially considering the deformation of elastomers with defect materials [41,42]. The deformation of defect materials is generally more obvious than that of normal non-damage, and there are complex nonlinear and discontinuous mutations in the interior. These aspects need to be further studied. There are also problems such as solid heat transfer, crystal heat conduction and material dislocation theory that can be referred to the classic works. Reference [43] can provide many professional insights.

### 1.3. Contributions

This paper mainly studies the deformation of 2D and 3D thin plates. We established a separate mechanical field, a separate temperature field and a thermal-mechanical coupling multi-physical model. This paper has five contributions: Our first contribution is that we analyze the causes of plate deformation, which is related to Young’s modulus *E*, load *F*, plate length *L* and deformation factor α. In addition, through numerical comparison, it is found that the semi-analytical Fourier series method is more accurate, but the derivation is relatively complex. The second contribution is that a 2D transient heat conduction model of graphene thin films with a special shape is established. In addition, three discrete schemes are given, the advantages and disadvantages of the three methods are analyzed. The 2D transient heat conduction equation is solved by the FDM-FEM method. The implicit Euler method to discrete the time can improve numerical stability, and the FEM is used to discrete the space term. The third contribution of this paper is to compare the differences between adiabatic conditions and convective conditions by using graphical method and curve trend. The fourth contribution is that we established a multi-physics coupling model of mechanical -temperature field. We propose a 3D-DTMCM model of single crystal silicon thin plate heated by laser with periodic motion characteristics. Meanwhile, we give the formula where the temperature radiation of laser heat source satisfies the Gauss distribution characteristics. The temperature difference of single crystal silicon thin plate under 3D adiabatic condition and natural convection condition are compared. In the coupling model, we found that the higher the temperature was, the higher the strain energy of the thin plate would be. Finally, the fifth contribution is that we propose a deformation model of the material plate with defects is established in Section 5, and we also consider comprehensive factors such as high temperature, load, internal mixed cracks, etc.

### 1.4. Structure and Framework of This Paper

The framework and content layout of this paper is composed of five sections. Section 2 mainly introduces two models of thin plate bending. In the rectangular coordinate system, this paper deduces the thin plate bending equation based on the energy method, and gives the numerical examples of the deformation of square plate and rectangular plate. Furthermore, the problem of circular plate deformation is studied. In the polar coordinate system, Fourier series is used as the approximation of the deflection function. The analytical function of the bending deformation of the circular plate is obtained, and the numerical example of the deformation of the circular plate is given. In a separate temperature field, Section 3 introduces the study of the numerical difference of 2D graphene sheet under different thermal boundary conditions. The FDM-FEM is used to discrete the 2D heat conduction equation, and the temperature changes of graphene films at different times are output. Section 4 studied the deformation of 3D single crystal silicon thin plate in the multi-physical field model of thermal coupling. We proposed a new dynamic thermal-mechanical coupling model (3D-DTMCM). The laser heating formula with periodic motion, and studied the relationship between temperature and strain energy. This paragraph description of the above article structure can help readers quickly understand the research focus of this paper. In Section 6, we also discussed the stress field distribution of mixed cracks under high temperature and high load. At last, the summary of the whole paper, highlighting our research contribution and the relevant conclusions of numerical experiments.

## 2. Bending Deformation Model of Thin Plate

Thin plate deformation is an important branch of elastic mechanics, and an accurate model can be used to analyze the deformation of elastic body, which can be applied to practical material assembly design, safety assessment of industrial construction, mechanical products and so on [44]. For the bending problem of thin plate, a fourth-order bending equation can be obtained by the energy method, which can describe the deformation process of thin plate. Of course, the deformation is limited to move within the two-dimensional level. If the three-dimensional deformation is considered, the shell model or the three-dimensional elastic equation should be established [45,46]. In this paper, we derive the differential equation of thin plate deformation, and analyze the important factors affecting deformation.

### 2.1. Classification of Plate and Deflection

The model generally needs to consider the basic Kirchhoff assumptions, the control equation of thin plate deformation, the boundary and the effect of thin plate deformation under different shapes and loads. Plate classification is generally based on the ratio of plate thickness to the minimum side (length or width). Specially thick plate and very thin plate, secondly, large deflection and small deflection corresponding to the physical model is different. At the same time, the ratio of deflection to plate thickness can determine which can determine the magnitude of the change. Small deflection is $\frac{w}{h_{\delta }}<\frac{1}{5}$, medium deflection: $1<\frac{w}{h_{\delta }}<5$, large deflection: $\frac{w}{h_{\delta }}\geq 5$. Therefore, it is also necessary to classify as material geometric size and deformation degree, the classification of plates and deflections is detailed in Table 1.

Of course, different deflections need to establish different physical models, and they also have different application scenarios. This paper provides the application range of common different deflections.

(1) The application range of small deflection.

The deformation range is relatively small, which can be generally used for the dust removal method of vibration, the auxiliary calculation of some buckling loads, and the calculation of small deflection of the recovery valve plate of automobile shock absorber. Based on the small deflection bending theory of thin plate, the buckling of shell and thin plate is analyzed. For the polar coordinate disk, the semi-analytical solution of the deflection equation can be obtained by using the generalized Fourier series solution.

(2) Application of mid-deflection.

The problem of mid-deflection calculation and modeling is encountered when the vehicle load is long-term acting on the bridge. The reasons for the formation of long-term deflection are analyzed. The cracks in the beam and the control of prestress play a key role. The numerical prediction of mid-deflection and the stability analysis of reinforced concrete under the action of mid-deflection are commonly used in the design of factory building structures.

(3) Large deflection application.

A large deflection of the elastic Timoshenko beam is a nonlinear PDE, and the soft soil tunnel analysis will be applied to the large deflection model. For the large deflection experiment of bridge, laser scanning can be used to obtain relatively accurate data. The small film material also has a large deflection phenomenon, such as the pressure sensor made of graphene. The numerical model belongs to the large deflection problem. With the increase of load, the elastic modulus and the number of graphene layers show a decreasing effect on the deflection deformation, but the natural frequency and modal characteristics still depend on the prestress, and are less affected by the film parameters.

### 2.2. Bending Model Theory of 2D Thin Plate

The deflection in this paper refers to the maximum deformation of the thin plate under load, the displacement along the vertical direction *y*. The displacement is commonly used to replace the stress tensor in the elastic equation, and the deflection is commonly used to replace the stress tensor in the thin plate bending model [47]. According to the assumption of straight normal, the straight line perpendicular to the midplane of the plate is still perpendicular to the midplane after bending, and the displacement component of the thin plate is recorded as:

(1) $$u={\left(u,v,w\right)}^{T}={\left(-z\frac{\partial w}{\partial x},-z\frac{\partial w}{\partial y},w\left(x,y\right)\right)}^{T}.$$

The generalized strain of thin plate bending is denoted as:

(2) $$\chi ={\left(\begin{array}{ccc} \chi_{x} & \chi_{y} & \chi_{xy} \end{array}\right)}^{T}={\left(\begin{array}{ccc} -\frac{\partial^{2}w}{\partial x^{2}} & -\frac{\partial^{2}w}{\partial y^{2}} & -2\frac{\partial^{2}w}{\partial x\partial y} \end{array}\right)}^{T}.$$

In addition, the out-of-plane stress component of the thin plate is much smaller than the in-plane component, and the stress of the plane can be expressed by using the strain according to the physical equation:

(3) $$\left\{\begin{array}{c} \sigma_{x}=-\frac{Ez}{1-v^{2}}\left(\frac{\partial^{2}w}{\partial x^{2}}+v\frac{\partial^{2}w}{\partial y^{2}}\right)\\ \sigma_{y}=-\frac{Ez}{1-v^{2}}\left(v\frac{\partial^{2}w}{\partial x^{2}}+v\frac{\partial^{2}w}{\partial y^{2}}\right)\\ \tau_{xy}=-\frac{Ez}{1+v}\frac{\partial^{2}w}{\partial x\partial y}. \end{array}\right.$$

Then, according to the three-dimensional equilibrium equation, we can get:

(4) $$\left\{\begin{array}{c} \tau_{xz}=\frac{Ez}{2\left(1-v^{2}\right)}\left(z^{2}-\frac{h^{2}}{4}\right)\frac{\partial }{\partial x}\nabla^{2}w\\ \tau_{yz}=\frac{Ez}{2\left(1-v^{2}\right)}\left(z^{2}-\frac{h^{2}}{4}\right)\frac{\partial }{\partial y}\nabla^{2}w\\ \sigma_{z}=-\frac{Eh^{3}}{6\left(1-v^{2}\right)}{\left(\frac{1}{2}-\frac{z}{h}\right)}^{2}\left(1+\frac{z}{h}\right)\nabla^{4}w. \end{array}\right.$$

The bending equation can be obtained according to the equilibrium relationship between bending moment and shear force (Appendix A) [48]. Of course, it can also be obtained according to the energy method, the total strain energy and external load along the deflection direction work sum; the specific derivation process is detailed in Appendix B [49,50]. This paper gives the form of the fourth order bending differential equation of thin plate, which is shown in Equation (5).

(5) $$\frac{\partial^{2}w}{\partial x^{4}}+2\frac{\partial^{4}w}{\partial^{2}x\partial^{2}y}+\frac{\partial^{4}w}{\partial y^{4}}=-\frac{q(x,y)}{D_{bend}}.$$

Therefore, the governing equation of bending deformation of elastic thin plate is a fourth-order bending equation, which is as follows:

(6) $$\nabla^{4}w=-\frac{q}{D_{bend}},$$

where $D_{bend}=\frac{Eh^{3}}{12\left(1-v^{2}\right)}$, *E* is the elastic modulus of the material, *h* is the thickness of the plate, *v* is the Possion’s ratio, and q(x,y) is the external force load. Equation (6) is the governing equation of bending deformation, the specific boundary conditions of Equation (6), there must be a specific known function w(x,y).

### 2.3. Boundary Conditions of Thin Plate Bending

For the deformation problem of a rectangular thin plate, it is necessary to specify at least two boundary conditions [51,52]. The numerical examples in Section 2 mainly consider the Free boundary (F) condition and Clamped boundary (C). In addition, there are two types of boundary conditions, which are the rolling boundary condition and simple support boundary conditions. These two boundary equations can reference the Appendix C for details.

(1) Clamped boundary (C).

If the edge of the plate x=a is fixed, the deflection and the rotation angle of the middle surface at the boundary are 0, that is,

(7) $$w_{x=a}=0,\;\frac{\partial w}{\partial x}=0.$$

(2) Free boundary (F).

If the elastic thin plate is completely free at x=a, then there is no bending moment, torque or shear, then it is satisfied that:

(8) $${\left.M_{xx}\right|}_{x=a}={\left.M_{xy}\right|}_{x=a}={\left.Q_{xz}\right|}_{x=a}=0.$$

According to the thin plate theory, these three boundary conditions can be combined and simplified into the following two boundary conditions, where $Q_{xz}+\frac{M_{xy}}{\partial y}$ is the effective shear, or Kirchoff shear, and there is no need to impose a free end boundary condition. Another equivalent expression is:

(9) $${\left.M_{xx}\right|}_{x=a}=0,\;Q_{xz}+{\left.\frac{M_{xy}}{\partial y}\right|}_{x=a}=0.$$

### 2.4. Example 1 2D Plates Deformation Simulation

Before solving the deformation of the 2D thin plate, we need to give the equation for the bending of the plate. As for the fourth-order bending differential equation of thin plate, this follows in Equation (10).

(10) $$\frac{\partial^{2}w}{\partial x^{4}}+2\frac{\partial^{4}w}{\partial^{2}x\partial^{2}y}+\frac{\partial^{4}w}{\partial y^{4}}=-\frac{q(x,y)}{D_{bend}}.$$

**Example** **1a.** *Considering a square rectangular metal plate, the edge length of the plate is a = 200 mm, the elastic modulus of the material E = 206 Gpa, and Poisson’s ratio is v = 0.3, assuming that the thickness of the thin plate h = 6 mm, the metal plate belongs to the isotropic material which satisfies the Kirchhoff assumption, and that the left side is fixed by the fixed boundary condition, which can be expressed as the following form:*

(11) $${\left.w(x,y)\right|}_{\Gamma_{1}}=0,{\left.w_{x}\left(x,y\right)\right|}_{\Gamma_{1}}=0.\;\Gamma_{1}=\left\{\left(x,y\right)|x=0,0\leq y\leq a\right\}.$$

The right end of the square metal sheet subjected to vertical downward external load.

(12) $${\left.q_{1}\left(x,y\right)\right|}_{\Gamma_{2}}=-2000N\;\Gamma_{2}=\left\{\left(x,y\right)|x=a,0\leq y\leq a\right\}.$$

**Example** **1b.** *Considering the bending deformation of a rectangular plastic strip (High pressure polyethylene), the elastic modulus of the material* E=0.15Gpa *, the isotropic materials, the boundary conditions of length b = 160 mm, width d = 30 mm are also fixed at the left end, the initial condition for deflection is Formula (14).*

(13) $${\left.w(x,y)\right|}_{\Gamma_{3}}=0,{\left.w_{x}\left(x,y\right)\right|}_{\Gamma_{3}}=0.\;\Gamma_{3}=\left\{\left(x,y\right)|x=0,0\leq y\leq d\right\}.$$

The right end is subjected to vertical upward force $q_{2}\left(x,y\right)$.

(14) $${\left.q_{2}\left(x,y\right)\right|}_{\Gamma_{4}}=800N\;\Gamma_{4}=\left\{\left(x,y\right)|x=b,0\leq y\leq d\right\}.$$

The deformation experiment for an elastic plate under the constant temperature T=25°C, and the stress σ of the plate and the variation of deflection w can be solved. Then, the deflection w(x,y) is used to represent the corresponding physical quantities such as displacement and stress. The model established in this paper is used to solve a harmonic equation of plate bending $\nabla^{4}w=-\frac{1}{D_{bend}}q$. Using the Galerkin numerical method to solve, and uses Q4 element to discrete the two thin plate.

Then, Equation (10) is discretized according to the Galerkin finite element method, and the shape function in the element is denoted as $\phi_{i}\left(x,y\right)$. The solving domain is denoted as Ω. The model is solved by Q4 element, each element has four nodes, that is, there are 12 degrees of freedom. Then the variational form of the original Equation (10) is converted into Equation (15):

(15) $$D_{bend}{\;\int \int \;}_{\Omega }\phi_{i}\left(x,y\right)\nabla^{4}wdxdy=-{\;\int \int \;}_{\Omega }\phi_{i}\left(x,y\right)q\left(x,y\right)dxdy.$$

The first right term of Equation (15) is further processed; by using the divergence theorem, we can obtain the Formula (16).

(16) $$\begin{array}{cc} D_{blend}{\;\int \int \;}_{\Omega }\phi_{i}\left(x,y\right)\nabla^{4}wdxdy= & D_{bend}\left[{\;\int \int \;}_{\Omega }\nabla \cdot \left(\phi_{i}\left(x,y\right)\nabla \right)\nabla^{2}wdxdy-\right.\\ \; & \left.{\;\int \int \;}_{\Omega }\nabla \phi_{i}\left(x,y\right)\cdot \nabla \left(\nabla^{2}w\right)dxdy\right]. \end{array}$$

Then, using the partial integration method and finishing the second item of Equation (16), Γ is the boundary of the solution domain, and n is the unit normal vector pointing to the solution domain. So, we get the Equation (17).

(17) $$\begin{array}{cc} {\;\int \int \;}_{\Omega }\nabla \phi_{i}\left(x,y\right)\cdot \nabla \left(\nabla^{2}w\right)dxdy= & -\oint_{\Gamma }n\cdot \nabla \phi_{i}\left(x,y\right)\nabla^{2}wdl-\\ \; & {\;\int \int \;}_{\Omega }\nabla^{2}\phi_{i}\left(x,y\right)\left(\nabla^{2}w\right)dxdy. \end{array}$$

Bring Equation (17) into Equation (16), then we can get Equation (18) by combining the Green formula.

(18) $$\begin{array}{cc} {\;\int \int \;}_{\Omega }\phi_{i}\left(x,y\right)\nabla^{4}wdxdy & =-\oint_{\Gamma }\phi_{i}\left(x,y\right)n\cdot \nabla \left(\nabla^{2}w\right)dl\\ \; & +\oint_{\Gamma }n\cdot \nabla \phi_{i}\left(x,y\right)\nabla^{2}wdl+{\;\int \int \;}_{\Omega }\nabla^{2}\phi_{i}\left(x,y\right)\left(\nabla^{2}w\right)dxdy. \end{array}$$

Finally, we can obtain the final Galerkin integral formula as follows.

(19) $$\begin{array}{cc} {\;\int \int \;}_{\Omega }\nabla^{2}\phi_{i}\left(x,y\right)\nabla^{2}wdxdy & =\oint_{\Gamma }\phi_{i}\left(x,y\right)n\cdot \nabla \left(\nabla^{2}w\right)dl\\ \; & -\oint_{\Gamma }n\cdot \nabla \phi_{i}\left(x,y\right)\nabla^{2}wdl-\frac{1}{D_{bend}}{\;\int \int \;}_{\Omega }\phi_{i}\left(x,y\right)q\left(x,y\right)dxdy. \end{array}$$

The transverse shear force vector is denoted as: $F_{ts}=D_{bend}\nabla \left(\nabla^{2}w\right)$, and the bending moment tensor is denoted as $M=M_{xx}+M_{yy}=D_{blend}\left(1+v\right)\nabla^{2}w$. Then, the above formula can become Equation (20).

(20) $$\begin{array}{cc} D_{bend}{\;\int \int \;}_{\Omega }\nabla^{2}\phi_{i}\left(x,y\right)\nabla^{2}wdxdy= & -\int_{\Gamma }\phi_{i}n\cdot F_{ts}dl+\\ \; & \frac{1}{1+v}\oint_{\Gamma }Mn\cdot \nabla \phi_{i}dl-{\;\int \int \;}_{\Omega }\phi_{i}\left(x,y\right)q\left(x,y\right)dxdy. \end{array}$$

The basis function $\phi_{i}\left(x,y\right)$ in the approximate term uses a quadratic bilinear form function, which is converted to $N_{i}\left(\xi ,\eta \right)$ in the local coordinate system. Then the deflection approximate function can be written as a form function in $w\left(x,y\right)=\sum_{i=1}^{N_{e}}w_{i}N_{i}\left(\xi ,\eta \right)$. The form function is Q4 element with four nodes, the basis function is as follows:

(21) $$N_{i}\left(\xi ,\eta \right)=\frac{1}{4}\left(1+\xi_{i}\xi \right)\left(1+\eta_{i}\eta \right)\;\left(i=1,2,3,4\right).$$

Then, the linear equation can be obtained:

(22) $$K_{ij}^{e}w_{i}^{e}=F_{i}^{e}=\frac{1}{D_{bend}}q_{i},\;K_{ij}^{e}={\;\int \int \;}_{\Omega^{\varepsilon }}\nabla^{2}N_{i}\nabla^{2}N_{j}dxdy.$$

The bending stiffness matrix of the linear equation element is denoted as:

(23) $$K_{ij}^{e}={\;\int \int \;}_{\Omega^{e}}\nabla^{2}N_{i}\nabla^{2}N_{j}dxdy.$$

The right hand items of algebraic equations can be written as:

(24) $$F_{i}^{e}=-\frac{1}{D_{bend}}{\;\int \int \;}_{z_{i}}N_{i}q_{i}dxdy.$$

Finally, the right term $F_{i}^{e}=-\frac{1}{D_{bend}}{\;\int \int \;}_{z_{i}}N_{i}q_{i}dxdy$, and the algebraic equation is assembled into the entire bending moment stiffness matrix. A new linear equation group $A_{ij}w_{i}=b_{i}$ is formed. According to the corresponding relationship between the submatrix and the subscript of the total stiffness matrix, it can be converted into:

(25) $$A_{ij}=\sum_{i=1}^{N}K_{ij}^{e},\;b_{i}=\sum_{i=1}^{N}F_{i}^{e}.$$

In Equation (25), *N* represents the total number of elements, and $E_{i}$ represents the *i*-th element. A is the combination of all element stiffness matrices, and F is the right-hand term. Finally, only one large sparse matrix needs to be solved.

**Figure 1:**
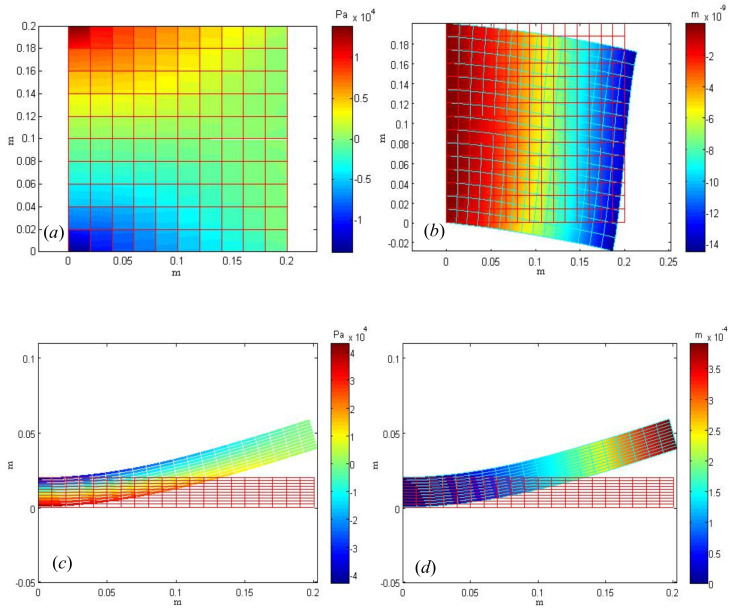
Numerical solution of thin plate deformation by Q4 element: (**a**). The stress $\sigma_{xx}$ of steel plate on mesh 1. (**b**) The displacement *V* of steel plate on mesh 2. (**c**) The stress $\sigma_{xx}$ of plastic materials with the deformation. (**d**) Displacement *V* of plastic materials.

Two deformation numerical examples are solved by the model established in this paper. The numerical results of Example 1a are Figure 1a,b with a bending model of a thin square plate. Meanwhile, the numerical results of Example 1b are Figure 1c,d. They are under the constant temperature environment deformation (without considering temperature). Due to different material parameters, the deformation of the plastic plate is larger than that of the steel plate. Figure 1a is a square plate of steel plate stress $\sigma_{xx}$ on mesh scale 10×10, the deformation magnification factor η=100. Figure 1b is the displacement *V* of the steel plate on the scale mesh 15×15, deformation amplification factor $\eta =2\times 10^{6}$. Figure 1c is the stress diagram of the plastic rectangular plate. It can be seen that there is stress concentration at the fixed end, and the mesh scale is 20×10. For Figure 1d, where the displacement variation in the *y* direction of the rectangular elastic plate. Here, it should be emphasized that our red mesh is the initial mesh, and the green is the deformation mesh. There is a scale factor in the program, which can adjust the deformation according to the need.

### 2.5. Deformation Model of Circular Plate in Polar Coordinates

#### 2.5.1. Theory of Circular Plate Bending

Considering the deformation of the circular plate under normal load, the polar coordinate (r,θ) can be used for modeling, we convert Cartesian coordinate system to polar coordinate system, the specific formulas are as follows:

(26) x=rcosθ,y=rsinθ.

Radius *r* is the distance from the center of the circular plate, and θ is the polar angle of counterclockwise rotation [53,54]. Using the standard coordinate transformation formula, according to the second-order partial derivative, there are:

(27) $$\frac{\partial^{2}w}{\partial x^{2}}=\cos^{2}\theta \frac{\partial^{2}w}{\partial r^{2}}-\frac{\sin2\theta }{r}\frac{\partial^{2}w}{\partial r\partial \theta }+\frac{\sin^{2}\theta }{r}\frac{\partial w}{\partial r}+\frac{\sin2\theta }{r^{2}}\frac{\partial w}{\partial \theta }+\frac{\sin^{2}\theta }{r^{2}}\frac{\partial^{2}w}{\partial \theta^{2}}$$

(28) $$\frac{\partial^{2}w}{\partial y^{2}}=\sin^{2}\theta \frac{\partial^{2}w}{\partial r^{2}}+\frac{\sin2\theta }{r}\frac{\partial^{2}w}{\partial r\partial \theta }+\frac{\cos^{2}\theta }{r}\frac{\partial w}{\partial r}-\frac{\sin2\theta }{r^{2}}\frac{\partial w}{\partial \theta }+\frac{\cos^{2}\theta }{r^{2}}\frac{\partial^{2}w}{\partial \theta^{2}}$$

(29) $$\frac{\partial^{2}w}{\partial x\partial y}=\frac{1}{2}\left(\sin2\theta \frac{\partial^{2}w}{\partial r^{2}}+2\frac{\cos2\theta }{r}\frac{\partial^{2}w}{\partial r\partial \theta }-\frac{\sin\theta }{r}\frac{\partial w}{\partial r}-2\frac{\cos2\theta }{r^{2}}\frac{\partial w}{\partial \theta }-\frac{\sin^{2}\theta }{r^{2}}\frac{\partial^{2}w}{\partial \theta^{2}}\right).$$

Then Formula (30) can be obtained by applying Laplace operator to act on the deflection function w(r,θ).

(30) $$\nabla^{2}w=\frac{1}{r}\frac{\partial }{\partial r}\left(r\frac{\partial w}{\partial r}\right)+\frac{1}{r^{2}}\frac{\partial^{2}w}{\partial \theta^{2}}.$$

Since the bending of thin circular plates also conforms to the fourth-order differential equation of bending in polar coordinates, it is necessary to apply the biharmonic operator to the deflection function w(x,y).

(31) $$\nabla^{4}w=L\left(w\right)+\frac{2}{r^{2}}\frac{\partial^{4}w}{\partial r^{2}\partial \theta^{2}}-\frac{2}{r^{3}}\left(\frac{\partial^{3}w}{\partial r\partial \theta^{2}}\right)+\frac{4}{r^{4}}\frac{\partial^{2}w}{\partial \theta^{2}}+\frac{1}{r^{4}}\frac{\partial^{2}w}{\partial \theta^{4}}.$$

The specific form of the *L* function in the formula is as follows:

(32) $$L\left(w\right)=\frac{1}{r}\frac{\partial }{\partial r}\left[r\frac{\partial }{\partial r}\left(\frac{1}{r}\frac{\partial }{\partial r}\left(r\frac{\partial }{\partial r}\right)\right)\right]=\frac{\partial^{4}w}{\partial r^{4}}+\frac{2}{r}\frac{\partial^{3}w}{\partial r^{3}}-\frac{1}{r^{2}}\frac{\partial^{2}w}{\partial r^{2}}+\frac{1}{r^{3}}\frac{\partial w}{\partial r}.$$

The analytical solution of the Fourth-Order deflection equation in polar coordinates can be solved semi-analytically method of Fourier series approximation, and the detailed solution process and derivation, please refer to Appendix D [55,56,57]. Among them, *a* is the radius of the plate, constant $A_{i},B_{i},C_{i}$ and $D_{i}$ determined by boundary conditions, when the load density function *f* is uniform on the whole circular plate, there is a suitable special solution:

(33) $$w\left(r,\theta \right)=-\frac{f(r,\theta )}{64D_{bend}}r^{4}=-\frac{3\left(1-v^{2}\right)f\left(r,\theta \right)}{16Eh^{3}}r^{4}.$$

The corresponding second-order homogeneous general solution is:

(34) $$w\left(r,\theta \right)=-\frac{f(r,\theta )}{64D_{bend}}{\left(a-r\right)}^{2}=-\frac{3\left(1-v^{2}\right)f\left(r,\theta \right)}{16Eh^{3}}{\left(a-r\right)}^{2}.$$

The following contents will provide two specific numerical examples to solve the bending problem of circular plate. The semi-analytical Fourier series method and the other selective finite element numerical method are used respectively. We also compare the advantages and disadvantages of these two methods.

#### 2.5.2. Circular Plate Bending Numerical examples

The geometry size of thin circle steel disc selected in this example: radius of disc is *a* = 1 m, thickness *h* = 0.05 m, Young’s modulus *E* = 206 Gpa, Poisson’s ratio *v* = 0.3, density $\rho =7.9\times 10^{3}\;\mathrm{kg}/m^{3}$. So the bending Equation (35) is to be solved.

(35) $$\nabla^{4}w=-\frac{q}{D_{bend}}=-\frac{12\left(1-v^{2}\right)q}{Eh^{3}}.$$

The side of the circular thin plate is fixed, the displacement boundary conditions are:

(36) $${\left.u(r,\theta ,z)\right|}_{\Gamma }={\left.v(r,\theta ,z)\right|}_{\Gamma }={\left.w(r,\theta ,z)\right|}_{\Gamma }=0$$

(37) $$\Gamma =\left\{\left(r,\theta ,z\right)|r_{i}=a,\theta_{i}=\frac{2\pi i}{n},i=1,2,\ldots n,0\leq z_{i}\leq h\right\}.$$

Normal load $q\left(x,y\right)=-400\sqrt{\left(x^{2}+y^{2}\right)}\pi N$, perpendicular to the upper surface of a thin plate, we convert it to the corresponding surface force load in polar coordinates as the formula.

(38) $${\left.q(r,\theta ,z)\right|}_{\Gamma_{s}}=-4\pi a\times 10^{2}N.$$

The surface area $\Gamma_{S}$ under normal load is:

(39) $$\Gamma_{S}=\left\{\left(r,\theta ,z\right)|0\leq r\leq a,\theta_{i}=\frac{2\pi i}{n},z=h\right\}.$$

The difference between Examples 1a and 1b is that the loading method is different. In this Example, the direction of external force load is parallel to the plate, and it can also be understood as loading external force load tangentially along the right boundary of the rectangle. The direction of external force load in this example is along the normal direction of the plate plane, that is, perpendicular to the plate plane.

**Figure 2:**
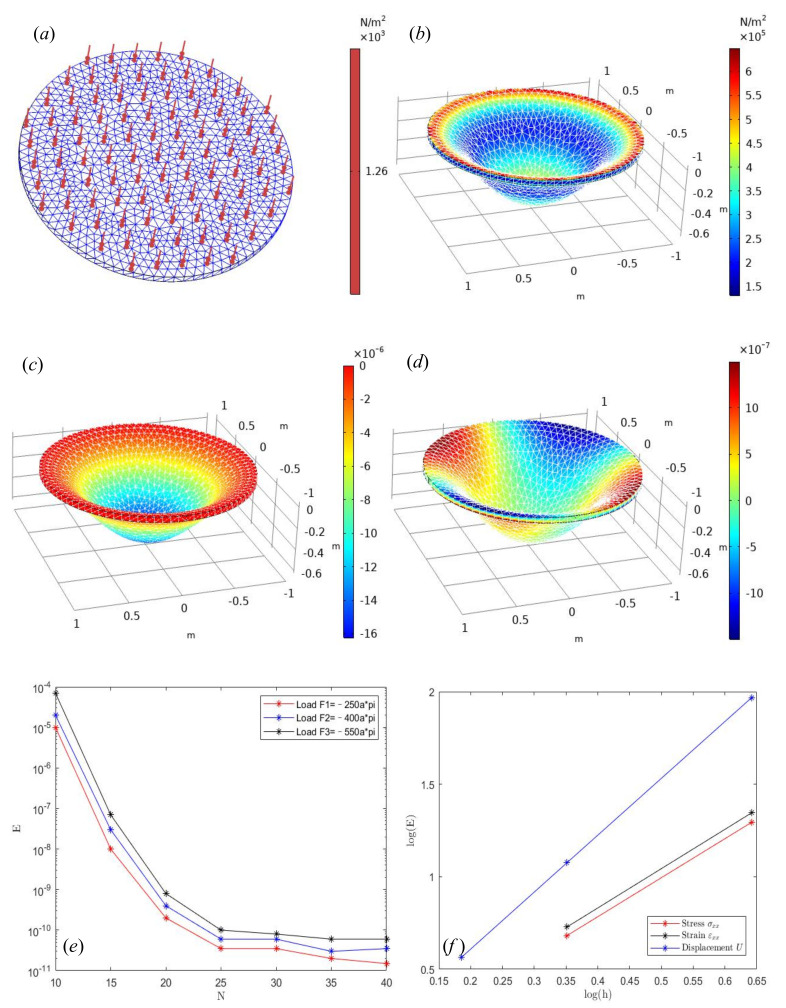
FEM numerical results of thin plate bending deformation in polar coordinates. (**a**) Circular plate region division and surface vertical load addition. (**b**) Von Mises stress of circular plate. (**c**) Displacement deflection function w(x,y). (**d**) The cloud map of strain $\varepsilon_{xy}$. (**e**) The semi-analytical solutions obtained by Fourier series under different loads. (**f**) Numerical convergence results using quadratic finite elements method.

The numerical results are as follows: Figure 2a is a diagram of mesh division and normal loads are added in the thin plate region. The thin plate region is discretized by quadratic element. The mesh vertex is 953, the number of elements is 3290, and the number of boundary elements is 1500. The red downward arrow indicates the direction of the external force load, and the density of the load region in the figure is uniform. Figure 2b is the Von Mises stress diagram. It can also be seen from the figure that the deflection function value at the boundary is w(x,y) = 0, and the variation of displacement at the center of the circular plate is the largest. Among them, the variable $\alpha =3.84\times 10^{5}$ is the deformation variable. Figure 2e is a semi-analytical solution, which can be obtained by Fourier series under three different loads. From the numerical results, it can be seen that with the increase of approximation terms *N* of Fourier series, the higher the approximation accuracy is.

The error is basically stable when the approximation term exceeds *N* = 25. In addition, the larger the load, the larger the error is slightly larger, mainly because the deflection equation applicable to the small deformation problem, when encountering medium deflection and large deflection, the solution error will be large. Figure 2f is the numerical convergence diagram of FEM. It can be seen that the basic convergence order of displacement *U* is basically close to 3, and the convergence order of stress $\sigma_{xx}$ and strain $\varepsilon_{xx}$ is basically close to 2. When the boundary element of the disk is more than 130, the error of the numerical solution of deflection is about $10^{-5}$. Compared with the semi-analytical method, the accuracy is relatively low, but the derivation process of the Fourier series method is relatively complex, which is also the comparison of the advantages and disadvantages of the two methods.

## 3. 2D Transient Heat Conduction Equation Solved by FDM-FEM

In order to study the variation law of internal temperature of two-dimensional elastic plate, this problem is equivalent to solving a 2D transient heat conduction equation. For some regular rectangular domains, they have analytical solutions, but this paper presents a special superposition region, and the analytical solution is difficult to obtain [58]. Therefore, we can only solve them by numerical methods. We give three numerical discretization schemes for FDM-FEM, and we choose the implicit Euler-FEM to solve the heat conduction problem of graphene film with different boundary conditions.

In order to study the adiabatic condition and temperature convection of plate problem. It is generally necessary to set the geometric solving domain and material parameters, and then make the mesh division of the geometric region. The selection of the mesh type is closely related to the characteristics of the solution domain. If the geometric region is a regular region, the mesh should choose uniform mesh as far as possible. If the calculation domain is an irregular region, it is necessary to use an adaptive mesh division. The reason is that the gradient value of the temperature field in the irregular region increases quickly, and the appropriate refinement of the mesh is beneficial to improve the numerical accuracy. Finally, it is due to the discretization of the heat conduction equation by FDM-FEM and the solution of the algebraic equation. In order to facilitate the comparison of the results, the numerical solution of the temperature field will be visualized. The following contents will give the theory of finite element combined with finite difference method for discretization of the two-dimensional transient heat conduction equation. The two-dimensional heat conduction equation can be expressed as:

(40) $$\rho c\frac{\partial T}{\partial t}=\frac{\partial }{\partial x}\left(k_{x}\frac{\partial T}{\partial x}\right)+\frac{\partial }{\partial y}\left(k_{y}\frac{\partial T}{\partial y}\right)+\rho q_{i}.$$

In Equation (40), ρ is the density (unit kg/m${}^{3}$), and *c* is the specific heat capacity of the material (unit J/kg · °C). The materials studied in this paper are isotropic materials, so the thermal conductivity coefficient is the same as $k=k_{x}=k_{y}$, $q_{i}$ is the heat source inside the material. In this paper, the material without heat source is selected, namely $q_{i}=0$. Therefore, the above equation can be abbreviated as:

(41) $$\rho cT_{t}=k\Delta T\;\left(x,y,t\right)\in \Omega \times T_{m}.$$

Three different temperature boundary conditions are expressed as follows:

(1) The first boundary condition:
  The temperature function of the boundary is a known function:
  (42) $${\left.T\right|}_{\Gamma_{1}}=T_{0}\left(x,y,t\right);$$
(2) The second boundary condition is the heat flux function:
  (43) $${\left.k\left(\frac{\partial T}{\partial n}\right)\right|}_{\Gamma_{2}}=q\left(x,y,t\right);$$
(3) Adiabatic boundary conditions
  (44) $${\left.k\left(\frac{\partial T}{\partial n}\right)\right|}_{\Gamma 3}+h\left(T-T_{m}\right)=q\left(x,y,t\right).$$

Among them, *h* is the heat exchange coefficient, the corresponding unit lis W/m${}^{2}$·K. $T_{m}$ is the environment temperature, *k* is the thermal conductivity. The above three cases contain the boundary conditions of different scenarios. The third type of boundary is also one of the focuses of our study. In addition, adiabatic conditions are applied in industrial insulation panels, insulation walls, cold chain vehicles, etc.

The solution domain of any element can be denoted as $\Omega^{e}$, and the temperature function of any point in the element is denoted as $T\left(x_{i},y_{i},t_{i}\right)$. If the triangular mesh is divided, the temperature of the three vertices of the triangle is $T_{i},T_{j},T_{m}$ in turn, and the space term and the time term are considered separately. The temperature function T(x,y,t) can be linearly expressed by the function value of the element vertices. The higher the order of basis function, the higher the accuracy of the numerical solution. If the finite element basis function is $\varphi_{1},\varphi_{2},\ldots ,\varphi_{m}$, the finite element numerical solution $T_{h}^{n}$ can be expressed by the linear combination of the basis function:

(45) $$T_{h}^{n}=\sum_{i=1}^{m}T_{i}^{n}\varphi_{i}=T_{1}^{n}\varphi_{1}+T_{2}^{n}\varphi_{2}+\ldots +T_{m}^{n}\varphi_{m}.$$

Similarly, we selected the test function to be denoted by $T^{*}\in H_{0}^{1}\left(\Omega \right)$ and it can also be linearly represented by the basis function:

(46) $$T_{h}^{*}=\sum_{j=1}^{m}T_{j}\varphi_{j}=T_{1}\varphi_{1}+T_{2}\varphi_{2}+\ldots +T_{m}.\varphi_{m}$$

Because Equation (41) is a time-dependent equation, we use finite difference method to discrete the time term, and then, using Galerkin finite element method to discrete the space term. In this paper, three numerical methods are given, which used to discrete the heat conduction time term, they are the Displaying Euler, Implicit Euler and Crank–Nicholson methods.

(1) Displaying Euler
  (47) $$\rho c\frac{T^{n+1}-T^{n}}{\Delta t}-k\Delta T^{n}=0.$$
(2) Implicit Euler method
  (48) $$\rho c\frac{T^{n+1}-T^{n}}{\Delta t}-k\Delta T^{n+1}=0.$$
(3) Crank–Nicholson method
  (49) $$\rho c\frac{T^{n+1}-T^{n}}{\Delta t}-\frac{1}{2}k\left(\Delta T^{n}+\Delta T^{n+1}\right)=0$$

Comparison of three numerical methods: Although Displaying Euler is relatively simple and easy to calculate, the implicit scheme is more stable. The accuracy of the explicit and implicit descriptions of the time term is first order, and the CN scheme is second order. The accuracy of the numerical solution of the spatial term is related to the selection of the basis function and to the smoothness of the accurate solution CN is unconditionally stable. In general, the spatial term distribution of temperature is the focus of our study, and the time term error should be within an acceptable range. Ultimately, the implicit Euler scheme is used to discrete the time term, and the weak scheme formed by Q4 element discretization for the spatial term. The discretization process is as follows:

According to the variational method, the two sides of the equation are multiplied by the test function $T^{*}\left(x,y\right)\in H_{0}^{1}\left(\Omega \right)$ at the same time, and the variables *x*, *y* integrals on the region Ω are obtained as follows:

(50) $$\rho c\int_{\Omega }\left(\frac{T^{n+1}\left(x,y\right)-T^{n}\left(x,y\right)}{\Delta t}-k\Delta T^{n+1}\left(x,y\right)\right)T^{*}\left(x,y\right)dxdy=0.$$

Using the first Green formula, combined with the second and third boundary conditions, it is easy to get the following Equation (51):

(51) $$\oint_{S}T^{*}\frac{\partial T}{\partial n}ds=\int_{\Omega }T^{*}\Delta Tdxdy+\int_{\Omega }\nabla T^{*}\nabla Tdxdy.$$

After moving the right term of Equation (51), we can obtain a new formula:

(52) $$\int_{\Omega }T^{*}\Delta Tdxdy=\oint_{S}T^{*}\frac{\partial T}{\partial n}ds-\int_{\Omega }\nabla T^{*}\nabla Tdxdy.$$

Equation (52) is simplified to:

(53) $$\begin{array}{cc} \; & \rho c\int_{\Omega }T^{n+1}\left(x,y\right)T^{*}\left(x,y\right)dxdy=\rho c\int_{\Omega }T^{n}\left(x,y\right)T^{*}\left(x,y\right)dxdy+\\ \; & \Delta tk\left(\oint_{s}T^{*}\frac{\partial T^{n+1}}{\partial n}ds-\int_{\Omega }\nabla T^{*}\nabla T^{n+1}dxdy\right). \end{array}$$

Then we use the matrix Equation (54) to represent differential Equation (53).

(54) $$MT^{n+1}+\Delta tkAT^{n+1}=g^{n}.$$

Among them, M and A are matrices, $g^{n}$ is the right-hand term. T is a vector, which need to be solved.

(55) $$M={\left[m_{ij}\right]}_{m\times m},\;m_{ij}=\rho c\int_{\Omega }\varphi_{i}\left(x,y\right)\varphi_{j}\left(x,y\right)dxdy.$$

(56) $$A={\left[a_{ij}\right]}_{m\times m},a_{ij}=\int_{\Omega }\nabla \varphi_{i}\left(x,y\right)\nabla \varphi_{j}\left(x,y\right)dxdy-\oint_{s}\varphi_{j}\left(x,y\right)qds.$$

The matrix form of the right term is $g^{n}={\left[g_{i}^{n}\right]}_{m\times 1},\;g_{i}=\rho c\int_{\Omega }T^{n}\left(x,y\right)\varphi_{j}\left(x,y\right)dxdy$, the unknown temperature variable is $T^{n+1}={\left(T_{1}^{n+1},T_{2}^{n+1},\ldots ,T_{m}^{n+1}\right)}^{T}$, with the above discrete theory we can easily solve the numerical solution of equation of heat conduction.

### Boundary Condition with Convection and Adiabatic of Graphene Films

This example mainly simulates the 2D transient thermal conduction of single layer graphene thin film material; graphene has a wide range of application: such as, supercapacitance (graphene battery has large storage capacity), flexible electronic screen (graphene has better conductivity), loudspeakers (graphene is ultra-thin, thickness is generally nanoscale, frequency sensitive, can produce high-quality sound), seawater purification (graphene has a good adsorption effect) [59,60]. In addition, graphene has good thermal conductivity, can be used as down jacket, heating carpet, fan coating heat dissipation effect is good. Graphene has also been known as the soft gold in the material field. Generally single layer film, double layer multilayer, and multilayer graphene is also widely used [61,62]. The battery capacity of graphene ball is 45% higher than that of lithium battery capacity. In addition, the application of nanostructured graphene tube in phonon heat conduction. The thermal conductivity of the object is also an important physical property of the material. Making good use of the heat transfer performance of graphene can save a lot of energy and enhance practicability. The above industrial applications reflect the potential research value of graphene materials.

This example simulates the temperature field distribution inside the film when the single-layer graphene material is subject to different boundary conditions. The geometric appearance of the graphene film designed in this example is two square nested patterns, and there is no overlapping area in the middle. It can also be seen as a regular shape with a side length of L=0.2m large, and then two small squares of equal size are dug at both ends of the diagonal. The side length of the small square is equal to a=0.065m. Several important material parameters will be encountered in solving the heat conduction equation of the graphene film, such as the constant pressure heat capacity $c_{\rho }$ = 2124 J/kg·K corresponding to the temperature of 300 K, and the density ρ is generally in $5-40\;\mathrm{kg}/m^{3}$. The density of this example is $\rho =40\;\mathrm{kg}/m^{3}$. The thermal conductivity *h* = 5000 W/m${}^{2}\cdot$K of monolayer graphene. Of course, if we want to calculate the deformation or mechanical analysis, we also need to understand the Young’s modulus *E* = 0.74 Tpa of monolayer graphene. The high strength graphene film (tensile strength is about 400 MPa) also has an in-plane negative Poisson’s ratio, and the Poisson’s ratio is at *v* = −0.25∼0.55. After understanding the basic physical parameters of graphene films, according to the law of heat transfer, the heat transfer law of monolayer graphene can also be described by two-dimensional transient heat conduction:

(57) $$\rho c\frac{\partial T}{\partial t}=\frac{\partial }{\partial x}\left(k\frac{\partial T}{\partial x}\right)+\frac{\partial }{\partial y}\left(k\frac{\partial T}{\partial y}\right).$$

The graphene solution region designed in this example has eight boundary conditions, involving three types of boundary conditions.

(1) Convection conduction:

The boundary $\Gamma_{1},\Gamma_{2},\Gamma_{3}$ has thermal convection conduction, and the external air temperature is T(x,y,t)=300K. The change of boundary conditions indicates that the boundary temperature of graphene film can be heat exchanged with the surrounding air, the boundary equation is as follows:

(58) $$q_{0}=h\cdot \left(T_{\mathrm{air}\;}\left(x,y,t\right)-T\left(x,y,t\right)\right)\;\left(x,y,t\right)\in \Gamma_{1},\Gamma_{2},\Gamma_{3}.$$

(2) Dirichlet boundary conduction:

Boundary $\Gamma_{2},\Gamma_{3},\Gamma_{6},\Gamma_{7}$ is the first boundary condition, which is equivalent to the known boundary temperature function $T_{0}\left(x,y,0\right)=1000\;K$.

(59) $$T\left(x,y,t\right)=T_{0}\left(x,y,0\right)\;\left(x,y,t\right)\in \Gamma_{2},\Gamma_{3},\Gamma_{6},\Gamma_{7}.$$

(3) Adiabatic boundary condition:

Boundary $\Gamma_{4}$ is an adiabatic boundary condition. That is, the internal temperature of graphene film is not thermal convection with the external air, the internal temperature is easy to maintain a high level.

(60) $$n\cdot \nabla T=\left(n_{x},n_{y}\right)\left(\frac{\partial T}{\partial x},\frac{\partial T}{\partial y}\right)=n_{x}\frac{\partial T}{\partial x}+n_{y}\frac{\partial T}{\partial y}=0.$$

**Figure 3:**
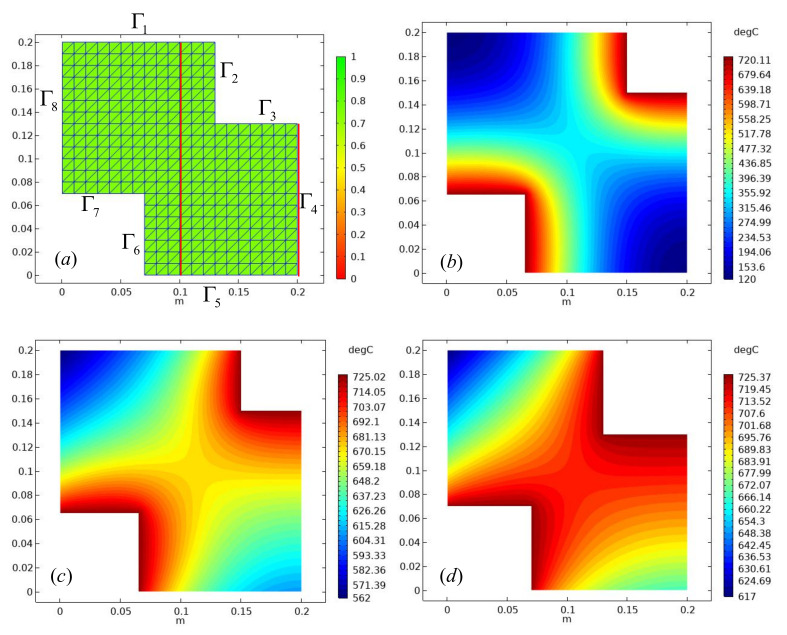

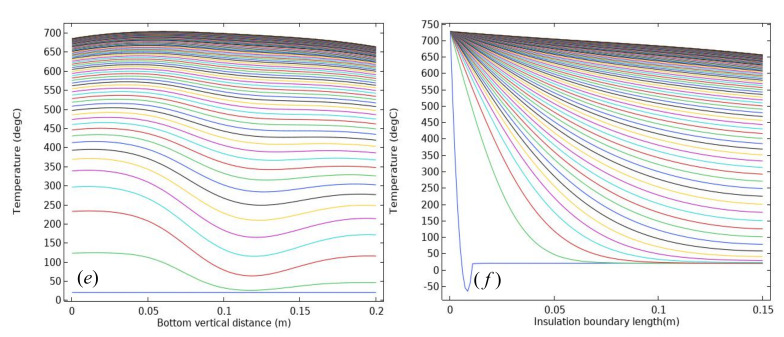
Numerical results of heat conduction of graphene films under different boundary conditions. (**a**) Uniform triangular mesh generation of graphene films. (**b**) Numerical cloud images of temperature at *t* = 0.045 s. (**c**) Numerical cloud map of temperature at *t* = 0.245 s (**d**) Numerical cloud map of temperature at *t* = 0.8 s. (**e**) Variation curve of distance and temperature on the vertical line at the bottom edge within 0–0.8 s. (**f**) Temperature variation curve of each point at the adiabatic boundary in 0–0.8 s.

According to the FDM-FEM numerical method given in this paper, the solution steps are also drawn according to the geometric region, and the material parameters are set. Then, the mesh is divided. This example uses the LST mesh element type, which can improve the accuracy of approximation. Compared with the CST element, the convergence speed of LST is faster. The standard details of mesh and boundary information are shown in Figure 3a; a total of 339 vertices, 600 triangular elements, 108 boundary elements, each element has 12 degrees of freedom, and the average mesh quality is η=0.92. The green mesh elements are all elements with good division effect. A total of eight boundaries of the single-layer graphene have been marked in clockwise direction. In this paper, the boundary conditions are not given symmetrically, so as to compare the difference between the adiabatic boundary condition and the convection boundary. Figure 3b shows the temperature numerical solution cloud of the graphene film at *t* = 0.45 s. It can also be seen from the figure that the boundary $\Gamma_{2},\Gamma_{3},\Gamma_{6},\Gamma_{7}$ belongs to the high temperature region, and the other boundaries belong to the low temperature region. The distribution of the initial temperature has good symmetry. The minimum value is 120 °C, and the maximum temperature is 720 °C.

When the two-dimensional transient heat conduction equation is used to solve the temperature change of graphene film, the temperature distribution functions corresponding to different time are different. When the time *t* = 0.245 s, we can see in Figure 3c a high-temperature region has gradually spread to the low-temperature region in the center of the film. At this time, we can obviously observe that the boundary $\Gamma_{4}$ and the boundary $\Gamma_{8}$ have no symmetry, and the temperature of the region near the boundary $\Gamma_{4}$ is higher. Figure 3d is the temperature field change diagram when *t* = 0.8 s. In this figure, only 0.8 s time can be seen, and the whole film temperature has been heated to about 600 °C. In addition, the adiabatic boundary condition $\Gamma_{4}$ has been significantly compared with the convective boundary condition $\Gamma_{8}$. We can see that the area near the adiabatic boundary is more likely to form a high temperature distribution. The reason is that there is no heat exchange and heat dissipation at the boundary $\Gamma_{4}$, so the energy is aggregated, so the temperature is higher. The thermal conductivity of graphene film and the special thermal boundary comparison we consider have not been studied. This reflects our research value, which is of great help to explore the thermal conductivity and application range of graphene.

## 4. Three Dimensional (3D) Dynamic Thermal-Mechanical Coupling Model (3D-DTMCM)

### 4.1. The Theories of 3D Dynamic Thermal-Mechanical Coupling Model

This example mainly considers the deformation of three-dimensional monocrystalline silicon non-metallic thin plate at different temperatures. Since monocrystalline silicon is the most important raw material in electronic products, it plays a huge role in chip manufacturing, semiconductors, solar panels and other aspects. This example mainly studies the influence of temperature on the deformation of monocrystalline silicon, which will change the internal material structure and mechanical properties of monocrystalline silicon semiconductor materials when they work at high temperature [63]. Therefore, in this regard, the theory has important industrial application value. It can promote the wide application of semiconductor materials.

The common wafers of monocrystalline silicon are 8 inches and 12 inches. In this example, a rectangular 3D monocrystalline silicon thin layer is given as the research object. The specific geometric parameters and material parameters are given below. For this rectangular monocrystalline silicon, the length is *L* = 3[in] = 0.0762 m, the width is *W* = 1[in] = 0.0254 m and the height is *h* = 275 um = 2.75 × 10${}^{-4}$ m. The purpose of this example is to analyze the deformation of single crystal silicon thin plate and the temperature variation of laser heating in the coupling model. Material parameters needed in the calculation of temperature change and mechanical properties are shown in Table 2.

In this numerical example, the change of temperature field is simply considered, and the single crystal silicon wafer is heated by a laser probe [64,65]. The heating process is divided into two cases. One is adiabatic with the surrounding air. In other words, there is no heat exchange. The other group of simulation experiments can exchange heat with air and there are energy losses and heat dissipation. The adiabatic simulation experiment needs to set a computational physical area in advance, that is, the internal heat does not thermal diffusion, and does not convection with the air. The heat energy is mainly concentrated in the computational physical area, and the heated laser moves $T_{\mathrm{laser}\;}=20$ s periodically around the middle line of the rectangular thin plate. In this paper, the process of laser heating for 60 s is simulated. The coordinates of the laser center point are expressed as $O\left(x_{\mathrm{laser}\;},y_{\mathrm{laser}}\right)$. The example assumes that $y_{\mathrm{laser}\;}=0\;m$, and the laser probe moves periodically when heating along the horizontal *x* axis direction. If the angular velocity w=2π, the motion period of the laser probe is $T_{\mathrm{laser}\;}=\frac{2\pi }{w}$, the amplitude *A* = 1, and the initial phase φ=0.5, then we can get the moving formula of transverse coordinates of heated laser which is as follows:

(61) $$\left.x_{\mathrm{laser}\;}=L\cdot S_{\mathrm{wave}\;}=L\cdot A{\left(-1\right)}^{\left\lfloor \frac{t}{T_{\mathrm{laser}\;}}+\varphi \right\rfloor }\right.\;t\in \left[0,60\right].$$

In addition, the temperature of the laser center is generally the highest, and the temperature heat flux of the surrounding coordinate points is Gaussian distribution. The radius of the laser radiation area is calculated as follows:

(62) $$\begin{array}{cc} R_{\mathrm{laser}\;} & =\sqrt{{\left(x-x_{\mathrm{laser}\;}\right)}^{2}+{\left(y-y_{\mathrm{laser}\;}\right)}^{2}}=\sqrt{{\left(x-x_{\mathrm{laser}\;}\right)}^{2}+y^{2}}\\ x & \in \left[0,L\right],y\in \left[-\frac{W}{2},\frac{W}{2}\right]. \end{array}$$

Laser temperature heat flux formula:

(63) $$\Phi_{q}=\frac{2P_{\mathrm{laser}\;}}{\pi R_{\mathrm{spot}\;}^{2}}e^{-\frac{2R_{\mathrm{laser}\;}^{2}}{R_{\mathrm{spot}\;}^{2}}},\;R_{\mathrm{laser}\;}\geq 0.$$

The following 3D transient heat conduction equation of single crystal silicon thin plate is given. Through this equation, the temperature change at any point in the solution domain can be calculated [66,67]. The material thin plate is assumed to be the isotropic material, that is, the thermal conductivity along *x*, *y* and *z* directions is the same, which satisfies the $k_{x}=k_{y}=k_{z}=k$ relationship. So the heat conduction equation is:

(64) $$\rho c\frac{\partial T}{\partial t}+\nabla \cdot \left(-k\nabla T\right)-Q=0.$$

Further processing Equation (64), we can obtain the detailed Equation (65) as follows:

(65) $$\rho c_{v}\frac{\partial T}{\partial t}=\frac{\partial }{\partial x}\left(k\frac{\partial T}{\partial x}\right)+\frac{\partial }{\partial y}\left(k\frac{\partial T}{\partial y}\right)+\frac{\partial }{\partial z}\left(k\frac{\partial T}{\partial z}\right)+Q.$$

For Equation (65), ρ is the material density $\mathrm{kg}/m^{3}$, *c* is the specific heat capacity J/(kg·k) of the material, and the plate needs energy when it is heated. *Q* is the heat generated by the internal heat source. $k_{x},k_{y},k_{z}$ is the thermal conductivity W/(m·K) along the *x*, *y*, and *z* directions, respectively. $\frac{\partial T}{\partial x},\frac{\partial T}{\partial y},\frac{\partial T}{\partial z}$ represents the heat flowing along the *x*, *y* and *z* directions in a unit time, respectively. The heat transferred into the object is balanced with the heat transferred out of the object when the object is heated. In addition, the temperature field distribution of the solution domain Ω needs to meet certain boundary conditions.

(1) The initial temperature of solid surface is 298.15 K, which can be expressed as follows.

(66) $$T_{1}\left(x,y,z,t\right)=\bar{T}\left(x,y,z,t\right)\;T_{1}\left(x,y,z,t\right)\in \Gamma .$$

(2) Adiabatic conditions:

In this example, considering that the laser prohibits heat loss while heating the silicon sheet and does not allow heat loss around the sheet, we can consider limiting a computational physical area where h∈[5,25] is the natural convection and heat exchange coefficient of the air in the unit of $W/m^{2}\cdot K$, and convection boundary condition satisfies the following Equation (67):

(67) $${\left.k\left(\frac{\partial T}{\partial n}\right)\right|}_{\Gamma 3}+h\left(T-T_{\mathrm{air}\;}\right)=q\left(x,y,t\right).$$

The solving domain of monocrystalline silicon thin plate is denoted as $\Omega \times T_{m}$:

(68) Ω={(x,y,z)∣0≤x≤L,0≤y≤W,0≤z≤h},T∈[0,60].

The physical region of adiabatic calculation of single crystal silicon plate is set as $\Omega_{c}\times T_{m}$, the scope of the time term is $T_{m}\in$[0,60].

(69) $$\Omega_{c}=\left\{\left(x_{c},y_{c},z_{c}\right)|-\frac{L}{2}\leq x_{c}\leq \frac{3}{2}L,-\frac{W}{2}\leq y_{c}\leq \frac{3W}{2},-2h\leq z_{c}\leq 2h\right\}.$$

(3) If $E_{mi}=0.8$ represents the emissivity of monocrystalline silicon, the calculated heat flux in the physical region satisfies the expression:

(70) $$q_{0}=E_{mi}\cdot \Phi_{q}.$$

Then, this example also studied the deformation of single crystal silicon wafer at different temperatures under the same load, which is the varying elastic model is considered in the experiment. With the increase of temperature, the elastic modulus decreases, and then the deformation becomes larger. The three-dimensional transient elastic equation can be expressed as:

Stress σ is a three-dimensional stress tensor matrix, $F={\left(f_{x}\left(t\right),f_{y}\left(t\right),f_{z}\left(t\right)\right)}^{T}$ is the load of silicon thin plate along each component, the three-dimensional gradient operator can be expressed as $\nabla =\left(\frac{\partial }{\partial x},\frac{\partial }{\partial y},\frac{\partial }{\partial z}\right)$, then the three-dimensional transient mechanical equation, Equation (71), shows:

(71) $$\rho \left(t\right)\frac{\partial^{2}u}{\partial t^{2}}=\nabla \cdot \sigma +F,\;u\in \Omega \times \left[0,T_{m}\right].$$

The Cauchy stress tensor introduced by three-dimensional deformable solid is expressed as:

(72) $$\sigma =\left(\begin{array}{ccc} \sigma_{xx} & \sigma_{xy} & \sigma_{xz}\\ \sigma_{yx} & \sigma_{yy} & \sigma_{yz}\\ \sigma_{zx} & \sigma_{zy} & \sigma_{zz}. \end{array}\right)$$

For three-dimensional stress tensor and strain there is the following relationship:

(73) $$\sigma_{ij}=\delta_{ij}\lambda \nabla \cdot u+2\mu \varepsilon_{ij}=\frac{\partial u_{k}}{\partial x_{k}}\lambda \delta_{ij}+\mu \left(\frac{\partial u_{i}}{\partial x_{j}}+\frac{\partial u_{j}}{\partial x_{i}}\right),$$

where *E* is the Young’s modulus of elastomer, *u* is Poisson’s ratio, and the Lamé constant formula is:

(74) $$\lambda =\frac{Ev}{\left(1+v)(1-2v\right)}.$$

The relationship between the three-dimensional stress tensor and strain is σ=Dε, where D is an elastic matrix, and the final stress can be expressed in the form of displacement. It should be noted that in the thermal-mechanical coupling model, the thermal stress and thermal strain generated in the high temperature environment cannot be ignored. The thermal strain is mainly related to the expansion coefficient α, and the three-dimensional thermal strain can be expressed as:

(75) $$\varepsilon_{T}=\left(1+v\right)\left(\begin{array}{c} \alpha T\\ \alpha T\\ \alpha T. \end{array}\right)$$

The calculation formula of thermal stress is as follows:

(76) $$\sigma_{T}=DBd-D\varepsilon_{T}=DBNq-D\varepsilon_{T}.$$

The elastic modulus is a variable about temperature, which can be determined by the energy method. There are usually two methods to calculate Young’s modulus, energy method and stress-strain method [68,69]. In this paper, the energy method is used to calculate Young’ s modulus. When the object is deformed, the total strain energy *E* of the object is calculated as follows:

(77) $$\begin{array}{c} E=\frac{1}{2}\;\int \int \int \;\left(\sigma_{x}\varepsilon_{x}+\sigma_{y}\varepsilon_{y}+\sigma_{z}\varepsilon_{z}+\tau_{\mathrm{yz}}\gamma_{\mathrm{yz}}+\tau_{zx}\gamma_{2x}+\right.\\ \left.\tau_{\mathrm{xy}}\gamma_{\mathrm{xy}}\right)dxdydz. \end{array}$$

It can be seen from Equation (77) that the total strain energy is equal to the sum of the product of stress and strain in each direction. If the stress and strain in a certain direction are 0, the strain energy along this direction is 0. The total energy of the object is equal to the sum of the initial energy $E_{0}$ and the energy after deformation, and this relationship can be written as:

(78) $$E=E_{0}+E\left(\varepsilon_{y}\right).$$

Usually, the uniform load is loaded along a certain direction in the experiment. For example, we assume that a uniform load ${\bar{\sigma }}_{y}$ is loaded along the *y* direction. Due to the Poisson effect, the corresponding strain $\varepsilon_{x},\varepsilon_{z}$ will also be generated in the *x* and *z* directions, but the contribution of deformation energy is derived from the Y direction. In Equation (79), $V={\;\int \int \int \;}_{\Omega }dxdydz$. Therefore, the above equation can be transformed into:

(79) $$\begin{array}{cc} \; & E\approx \frac{1}{2}\;\int \int \int \;\sigma_{y}\varepsilon_{y}dxdydz=\frac{1}{2}{\;\int \int \int \;}_{\Omega }E_{y}\varepsilon_{y}^{2}dxdydz=\frac{1}{2}E_{y}\varepsilon_{y}^{2}{\;\int \int \int \;}_{\Omega }dxdydz\\ \; & =\frac{1}{2}E_{y}\varepsilon_{y}^{2}V=E\left(\varepsilon_{y}\right). \end{array}$$

Then, the differential equations of Young’s modulus $E_{y}$ can be expressed as:

(80) $$E_{y}=\frac{1}{V}\frac{dE\left(\varepsilon_{y}\right)}{d\varepsilon_{y}^{2}}=\frac{L_{y0}^{2}}{V}\frac{d^{2}E\left(L_{y}\right)}{dL_{y}^{2}}.$$

In Equation (80), $L_{y}$ is the length along the y axis after denaturation, and $L_{y0}$ is the initial length.Then, according to the molecular dynamics method in Material Studio, we can study the variation characteristics of the Young’s modulus *E* along the *y* and *z* directions of the thin film with the temperature between 100 K and 600 k. In the NVT calculation system (let the number of simulated particles, the calculated volume, and the temperature of the system remain unchanged), the strain in a certain direction is simulated, and the strain $E\left(\varepsilon_{i}\right)$ after the stability of the system is counted. Combined with Equation (78), we can calculate Young ’ s modulus under the temperature:

(81) $$\left\{\begin{array}{c} E_{y}=179.33-6.8\times 10^{-3}T\;T\in \left[0,600K\right]\\ E_{z}=153.147-6.95\times 10^{-3}T. \end{array}\right.$$

### 4.2. Three Dimensional (3D) Single Crystal Silicon Sheet Deformation by the DTMCM Method

**Figure 4:**
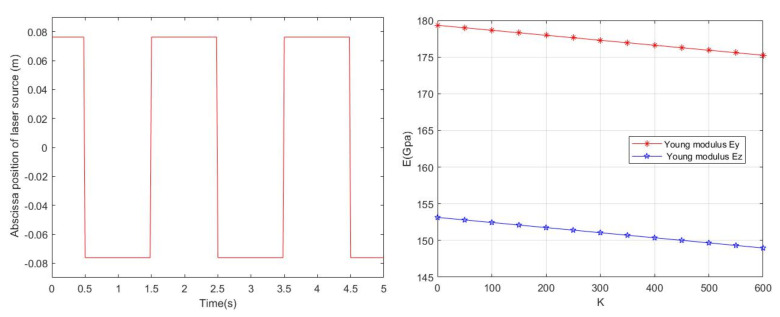
Laser Moving Period and Young’s Modulus Function of Monocrystalline Silicon Thin Plate. (**a**) Position where the transverse coordinates of the laser source remain with the periodic function (**b**) Relationship between Young ’s modulus and temperature.

According to the above theory, we can obtain the temperature and deformation of the single crystal silicon thin plate. This physical coupling model belongs to one-way coupling, that is, the increase of temperature has a great influence on the deformation of the material thin plate, but the temperature change caused by the deformation can be ignored. The temperature affects the deformation mainly through the elastic modulus, and the thermal strain is related to the expansion coefficient of the material [70]. At present, our model mainly considers the influence of temperature on the Young’s modulus, and then indirectly affects the deformation. The following Figure 4a is the position of the laser abscissa staying with the periodic function, which has obvious periodicity. Similar to the reciprocating motion on the central line of the rectangular plate, the temperature near the laser source satisfies the Gaussian distribution, that is, the temperature on the central line is the highest, which can also be reflected in the cloud picture of the numerical solution. Figure 4b shows the relationship between Young’s modulus and the temperature of the single crystal silicon thin plate. The numerical calculation results show that Young’s modulus decreases with the increase of temperature, and Young’s modulus in the z direction is 25 Gpa smaller than that in the y direction as a whole. This also indicates that the longitudinal direction is more likely to produce a large deformation than the transverse direction. The temperature change of $E_{y}$ or $E_{z}$ is 600 K. Young’s modulus changes about 5 GMpa, relative to steel materials this deformation resistance is relatively good.

**Figure 5:**
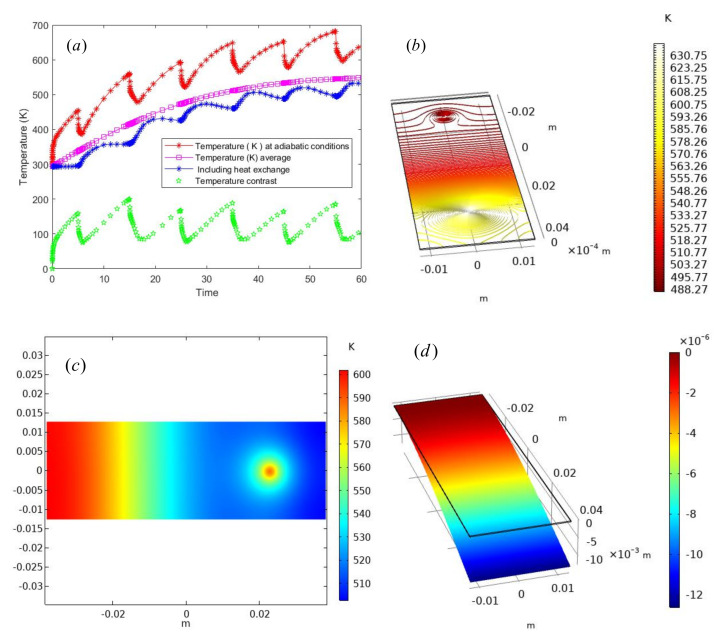

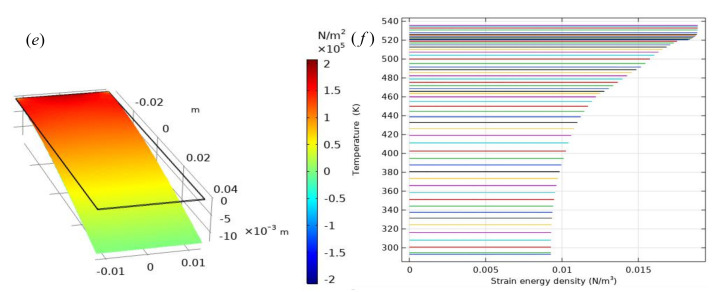
Numerical results of temperature field and force field coupling. (**a**) Comparison of temperature numerical solutions of laser heating under adiabatic and heat exchange conditions; (**b**) Isothermal line diagram of three-dimensional monocrystalline silicon at *t* = 30 s; (**c**) is the schematic diagram of temperature diffusion on the surface of monocrystalline silicon and laser power heating; (**d**) is the displacement nephogram along the *z* direction and the comparison diagram of deformation position and initial position; (**e**) is the stress nephogram along the *x* direction; (**f**) is the variation diagram of temperature and strain energy density.

Through the three-dimensional transient heat conduction equation established above, combined with the geometric parameters and material parameters of single crystal silicon material, the corresponding temperature and shape variables at different times are calculated. Finally, the numerical results of thermodynamic coupling calculation are shown in Figure 5, Figure 5a is a laser heating process with periodic motion characteristics alone. The red trend line represents the temperature change near the probe under the condition of adiabatic environment, and the local fluctuation is in an upward trend as a whole, and the maximum temperature is close to 700 K and the minimum temperature is close to 300 K at room temperature. The blue curve is heat loss. There is heat exchange and convection between the silicon thin plate and the surrounding air during laser heating, so the temperature is much lower than the adiabatic temperature. The maximum temperature is 500 K, and the pink line is the mean value of the temperature of the model, which is mainly the reference datum line. There is no practical significance. The green line is the temperature difference between the two models, and the fluctuation range is consistent with the results, indicating that the calculation of the model is relatively stable. Figure 5b is the isothermal line of laser heating when *t*= 30 s, and the two temperature vortices are mainly the reciprocating motion of the probe. The temperature did not form uniformly in time. Figure 5c shows the surface temperature change of the three-dimensional monocrystalline silicon thin plate at *t* = 45. It can also be seen from the figure that the laser heating probe is at the right end of the thin plate, and the thermal diffusion phenomenon is occurring in the high temperature region at the left end.

Then, the coupling model of temperature and force field is considered in our numerical experiment to study the influence of temperature on the deformation. The numerical implementation limits the single crystal silicon thin plate to be fixed for one section, and loads 100 N downward force for one section. Then, the temperature ranges from 300 K to 600 K. By increasing the temperature around the thin plate, the Young’s modulus *E* of the material will be directly affected. According to the numerical conclusion mentioned above, the Young ‘s modulus *E* will decrease with the increase of temperature, and then the deformation of the thin plate will also change differently. Meanwhile, Figure 5d is the displacement nephogram of the single crystal silicon thin plate in the z direction, which can be seen from the figure. The fixed end is the deepest red end, and the other side is loaded with external force. The deformation process is multiplied by the amplification factor α=1000 on the original displacement field. According to the displacement, the node coordinates are adjusted proportionally, and finally the deformation position is presented. Figure 5e is the stress $\sigma_{xx}$ of the thin plate along the *x* direction, which acts on the stress field with the same amplification factor α=1000. The black border above the thin plate is the initial position, and the support force that reflects the fixed is relatively large in the color map. Figure 5f is the relationship between the strain energy density and the temperature change. The conclusion can be obtained from the Figure 5f, the strain energy density increases with temperature, and it also reflects the increase of deformation. These conclusions are consistent with the conclusion that the elastic modulus *E* increases and the deformation increases in theory. The above numerical realization also reflects the idea of the coupling of temperature field and solid mechanics, the advantages of simulation experiments can reduce the cost and quickly simulate the material.

## 5. Discussion

### 5.1. Stress Model for Temperature Damaged and Microscopic Defects Elastic Plates

Under the combined action of loading conditions and environmental factors, micro-holes, micro-cracks and other micro-defects will occur in the material plate. In the continuous damage mechanics theory, the representative volume element (RVE) is taken as the research object, and it is considered that the total cross-sectional area in a certain direction of RVE is denoted as S, and the total micro-defect area is $S_{D}$. Then the damage degree is defined as the ratio of the total micro-defect area to the total cross-sectional area, namely, $D=\frac{S_{D}}{S}$. When the material is in a non-destructive state, $S_{D}=0$, D=0, and when $S_{R}=$ S, the material is completely destroyed, that is, the fatigue crack initiation is considered.

In the high temperature environment, the internal crystal structure of the sheet material deviates from the microscopic region of the complete lattice arrangement, forming point defects, line defects and surface defects [71,72,73]. *c* is the equilibrium concentration of interstitial atoms at high temperatures will change the grain boundary energy, different grain boundary energy corresponding to different grain boundary damage degrees, the damage degree of material caused by temperature can be represented by the volume fraction of grain boundary damage.

(82) $$\Delta A=c\exp\left(\frac{\Delta Ev}{k\left(T\left(t\right)-T_{0}\left(t\right)\right)}\right).$$

Then, we estimate the damage degree of temperature to the material by defining different grain boundary energy. γ(t) is the grain boundary energy at the current time. In the formula, it depends on the shear modulus *G* of the material, Poisson’s ratio *v* and the Birth’s vector b, A as integral constants, and depends on the atomic dislocation energy at the dislocation center. However, it is noted that the formula is only applicable to small angle grain boundaries, and does not apply to large angle grain boundaries. In fact, the grain boundaries of polycrystalline are generally large angle grain boundaries, and the orientation difference of each grain is mostly about 30–40 °C. The energy of large angle grain boundaries of various metals is about 0.25–1.0 J/m${}^{2}$. It has nothing to do with the orientation difference between grains.

(83) $$\gamma \left(t\right)=\frac{Gb\theta (\Delta A-\ln\theta )}{4\pi (1-v)},\;D=\frac{\gamma (t)}{\gamma_{\max}}.$$

The effective bearing area $S_{R}$ of RVE is defined as $S_{R}=\left(1-D\right)S$. Under the action of load *P* and the damage of temperature T(t), the effective stress $\tilde{\sigma }$ is:

(84) $${\tilde{\sigma }}_{T}=\frac{P}{S_{R}}=\frac{P}{(1-D)S}=\frac{\sigma }{1-D}.$$

In addition, the elastic modulus of damaged RVE is defined as $E_{D}=\left(1-D\right)E$, where E is the elastic modulus without damage; *E* is the elastic modulus with damage. The elastoplastic constitutive model used in this paper can consider isotropic and kinematic hardening [74,75]. In the case of small deformation, the total strain $\varepsilon_{ij}$ is decomposed into elastic strain $\varepsilon_{ij}^{e}$ and plastic strain $\varepsilon_{ij}^{p}$, namely $\varepsilon_{ij}=\varepsilon_{ij}^{e}+\varepsilon_{ij}^{p}$. Combined with the effective stress, the elastic strain of the material containing damage is as follows:

(85) $$\varepsilon_{ij}^{e}=\frac{1+v}{E}\left(\frac{\sigma_{ij}}{1-D}\right)-\frac{v}{E}\left(\frac{\sigma_{kk}}{1-D}\right)\delta_{ij},$$

(86) $$\varepsilon_{ij}^{e}=\frac{1+v}{E}\left(\frac{\sigma_{ij}}{1-D}\right)-\frac{v}{E}\left(\frac{\sigma_{kk}}{1-D}\right)\delta_{ij}.$$

Among them, *v* is Poisson’s ratio, $\delta_{ij}$ is the Kronecker product.

(87) $$F={\left(\frac{\sigma_{ij}}{1-D}-\alpha_{ij}\right)}_{eq}-\sigma_{y}$$

In addition, the yield function *F* of the damage coupling is:

(88) $${\tilde{\varepsilon }}^{p}=\tilde{\lambda }\frac{\partial F}{\partial \sigma_{ij}}=\frac{3}{2}\cdot \frac{\tilde{\lambda }}{1-D}\cdot \frac{{\left(\frac{\sigma_{kl}}{1-D}-\alpha_{kl}\right)}_{dev}}{{\left(\frac{\sigma_{kl}}{1-D}-\alpha_{kl}\right)}_{eq}},$$

(89) $$\tilde{p}=\sqrt{\frac{2}{3}{\tilde{\varepsilon }}_{ij}^{p}{\tilde{\varepsilon }}_{ij}^{p}}=\frac{\lambda }{1-D},$$

where the subscripts “eq” and “dev” represent the von Mises equivalent stress and the deviatoric part of the stress, respectively; $\alpha_{ij}$ is the back stress; $\sigma_{y}$ is the yield limit; $\tilde{\lambda }$ is the plastic multiplier; ${\tilde{\varepsilon }}^{p}$ is the plastic strain rate; $\tilde{p}$ is the cumulative plastic strain rate, the hardening law is as follows:

(90) $$\alpha_{ij}=\sum_{k=1}^{N}\alpha_{ij}^{\left(k\right)},$$

(91) $${\dot{\alpha }}_{ij}^{\left(k\right)}=\left(1-D\right)\left(\frac{2}{3}C_{k}\varepsilon_{ij}^{p}-\gamma_{k}\alpha_{ij}^{\left(k\right)}\tilde{p}\right).$$

In the above formula: *N* is the number of back stress components, $C_{k}$ and $\gamma_{k}$ are material parameters.

### 5.2. Stress Distributions for 2D Plane Crack Problems

Griffith, a British scientist, first applied the energy principle method to analyze the fracture of brittle materials such as glass and ceramics, and established the relationship between brittle stress, crack size and material properties, which laid the theoretical foundation of fracture mechanics. There are microcracks in brittle materials, and the stress concentration at the tip of microcracks greatly reduces the fracture strength of materials. For a certain size crack a, there is a certain critical stress $\delta_{c}$, when the external stress is greater than $\delta_{c}$, the crack propagation leads to fracture [76,77]. The condition of crack propagation is that the surface free energy required for propagation is provided by the elastic strain energy released by the system.

(1) Type I cracks (Opening displacement).

This crack model is established under the condition of tensile force δ>0, that is, only tensile stress can cause the opening-type propagation of the crack. We discuss the case of an infinite flat plate with a penetration crack of length 2a, and the two ends are subjected to a tensile stress δ perpendicular to the crack surface. At the distance *r* from the crack tip, the angle with the *x*-axis is θ. Taking a microfacet with size dx,dy at the θ according to the force balance, the normal stress at any point (r,θ) near the crack tip $\sigma_{xx},\sigma_{yy}$ and shear stress $\tau_{xy}$ are shown under below.

(92) $$\sigma_{xx}=\sigma \sqrt{\frac{a}{2r}}\cos\frac{\theta }{2}\left[1-\sin\frac{\theta }{2}\sin\frac{3\theta }{2}\right]$$

(93) $$\sigma_{yy}=\sigma \sqrt{\frac{a}{2r}}\cos\frac{\theta }{2}\left[1+\sin\frac{\theta }{2}\sin\frac{3\theta }{2}\right]$$

(94) $$\tau_{xy}=\sigma \sqrt{\frac{a}{2r}}\sin\frac{\theta }{2}\cos\frac{\theta }{2}\cos\frac{3\theta }{2}.$$

The stress can also be written as:

(95) $$\sigma_{ij}=\frac{K_{1}}{\sqrt{2\pi r}}\phi_{ij}\left(\theta \right),$$

where $K_{1}=\sigma \sqrt{\pi a}$, crack tip stress intensity factor.

(2) Type II cracks (Sliding displacement).

Three elements of fracture are crack size and shape, stress size (necessary) and fracture toughness of a material (material resistance) [78]. A measure of the ability of a material with cracks to resist fracture. The second type of crack has an intensity factor is $K_{2}=\tau \sqrt{\pi a}$. Then, the second type of crack stress field can be expressed as:

(96) $$\sigma_{xx}=\frac{-K_{2}}{\sqrt{2\pi r}}\sin\frac{\theta }{2}\left(2+\cos\frac{\theta }{2}\cos\frac{3\theta }{2}\right)$$

(97) $$\sigma_{yy}=\frac{K_{2}}{\sqrt{2\pi r}}\cos\frac{\theta }{2}\sin\frac{\theta }{2}\cos\frac{3\theta }{2}$$

(98) $$\tau_{xy}=\frac{K_{2}}{\sqrt{2\pi r}}\cos\frac{\theta }{2}\left(1-\sin\frac{\theta }{2}\cos\frac{3\theta }{2}\right).$$

The third type of crack has an intensity factor is $K_{3}=\tau \sqrt{\pi a}$. From elasticity–theoretical solutions for plane and spatial crack problems, approximation expressions can be developed that apply only in the immediate vicinity of the crack tip. If the polar coordinates *r* and φ at the crack tip. A series expansions for the crack tip stress fields are obtained with series terms that depend on the factor $r^{(n/2)-1}$ with n=1,2,3,… If only the first series term with $r^{-1/2}$ is considered. The formula of the mixed crack stress field (with i,j=x,y) in the two-dimensional plane is shown in Equation (99).

(99) $$\sigma_{\mathrm{ij}}=\frac{1}{\sqrt{2\pi \cdot r}}\left[K_{I}\cdot f_{\mathrm{ij}}^{I}\left(\varphi \right)+K_{\mathrm{II}}\cdot f_{\mathrm{ij}}^{\mathrm{II}}\left(\varphi \right)\right].$$

### 5.3. Stress Distributions for 3D Spatial Crack Problems

Type I and Type II are for plane cracks, and Type III for three-dimensional cracking problems. Similarly, the third type of crack belongs to tearing, and the corresponding crack stress fields are Equations (100) and (101), Type III cracks (Tearing displacement) are shown below.

(100) $$\tau_{xy}=\frac{-K_{3}}{\sqrt{2\pi r}}\sin\frac{\theta }{2}$$

(101) $$\tau_{yz}=\frac{K_{3}}{\sqrt{2\pi r}}\cos\frac{\theta }{2}.$$

In Cartesian coordinates, the following relations for the stresses $\sigma_{x},\sigma_{y},\sigma_{z},\tau_{xy},\tau_{xz}$, $\tau_{yz}$, are obtained:

(102) $$\sigma_{x}=\frac{K_{I}}{\sqrt{2\pi \cdot r}}\cdot \cos\frac{\varphi }{2}\cdot \left(1-\sin\frac{\varphi }{2}\cdot \sin\frac{3\varphi }{2}\right)-\frac{K_{\mathrm{II}}}{\sqrt{2\pi \cdot r}}\cdot \sin\frac{\varphi }{2}\cdot \left(2+\cos\frac{\varphi }{2}\cdot \cos\frac{3\varphi }{2}\right)$$

(103) $$\sigma_{y}=\frac{K_{I}}{\sqrt{2\pi \cdot r}}\cdot \cos\frac{\varphi }{2}\cdot \left(1+\sin\frac{\varphi }{2}\cdot \sin\frac{3\varphi }{2}\right)+\frac{K_{\mathrm{II}}}{\sqrt{2\pi \cdot r}}\cdot \sin\frac{\varphi }{2}\cdot \cos\frac{\varphi }{2}\cdot \cos\frac{3\varphi }{2}$$

(104) $$\tau_{\mathrm{xy}}=\frac{K_{I}}{\sqrt{2\pi \cdot r}}\cdot \sin\frac{\varphi }{2}\cdot \cos\frac{\varphi }{2}\cdot \cos\frac{3\varphi }{2}+\frac{K_{\mathrm{II}}}{\sqrt{2\pi \cdot r}}\cdot \cos\frac{\varphi }{2}\cdot \left(1-\sin\frac{\varphi }{2}\cdot \sin\frac{3\varphi }{2}\right)$$

(105) $$\tau_{xz}=-\frac{K_{\mathrm{III}}}{\sqrt{2\pi \cdot r}}\cdot \sin\frac{\varphi }{2},\;\tau_{yz}=\frac{K_{\mathrm{III}}}{\sqrt{2\pi \cdot r}}\cdot \cos\frac{\varphi }{2}$$

(106) $$\sigma_{z}=v\cdot \left(\sigma_{x}+\sigma_{y}\right)=\frac{2v}{\sqrt{2\pi \cdot r}}\cdot \left(K_{I}\cdot \cos\frac{\varphi }{2}-K_{\mathrm{II}}\cdot \sin\frac{\varphi }{2}\right).$$

In spatial mixed crack problems, including surface, edge and internal cracks, general loading can lead to overlapping of all three crack loading types [79]. In this case, the stress distributions with i,j=x,y,z in the vicinity of the crack can be represented in tensor notation as follows:

(107) $$\sigma_{\mathrm{ij}}=\frac{1}{\sqrt{2\pi \cdot r}}\left[K_{I}\cdot f_{\mathrm{ij}}^{I}\left(\varphi \right)+K_{\mathrm{II}}\cdot f_{\mathrm{ij}}^{\mathrm{II}}\left(\varphi \right)+K_{\mathrm{III}}\cdot f_{\mathrm{ij}}^{\mathrm{III}}\left(\varphi \right)\right].$$

As opposed to the plane solution, now an additional stress intensity factor $K_{\mathrm{III}}$ is included, which corresponds with crack loading type III and the function $f_{\mathrm{ij}}^{\mathrm{III}}\left(\varphi \right)$[80]. The $1/\sqrt{r}$-singularity of the stress field also applies to spatial crack problems (i.e., for $r\to 0,\sigma_{\mathrm{ij}}\to \infty$).

Furthermore, the crack tip stress intensity factors are defined by crack tip stress $\sigma_{yy}\left(r,0\right)$, $\tau_{yx}\left(r,0\right)$ and $\tau_{yz}\left(r,0\right)$ respectively:

(108) $$K_{1}=\lim_{r\to 0}\sqrt{2\pi r}\sigma_{yy}\left(r,0\right)$$

(109) $$K_{2}=\lim_{r\to 0}\sqrt{2\pi r}\tau_{yx}\left(r,0\right)$$

(110) $$K_{3}=\lim_{r\to 0}\sqrt{2\pi r}\tau_{yz}\left(r,0\right).$$

The establishment of the above model lays the foundation for the analysis of material plates with damage under high temperature. The final coupled stress field should be formed by the coupling of temperature damage stress, crack stress and elastic equation stress.

(111) $$\sigma_{\mathrm{coupling}\;}=\alpha_{1}\sigma_{T}+\alpha_{2}\sigma_{\mathrm{crack}\;}+\alpha_{3}\sigma_{\mathrm{elastic}\;}$$

(112) $$\sum_{i=1}^{3}\alpha_{i}=\alpha_{1}+\alpha_{2}+\alpha_{3}=1.$$

The main reason for the discussion in this theory is that the range of some parameters of the model need to be determined by experiments, so the obtained model is more worthy of promotion and is also in line with scientific rigor. However, the proposed coupling model theory provides a good foundation for the subsequent study of the deformation of the material plate with defects in the coupling field. The model can basically reflect two scales, the damage stress caused by temperature is established from the micro crystal, and the crack and elastic stress belong to the mesoscopic scale. We will further complete the numerical deformation of the material with defects in the future.

## 6. Conclusions

This paper is mainly focused on solving the bending deformation and thermal diffusion problems of 2D and 3D thin plates. For the 2D thin plates, we have derived the bending deformation equation of rectangular and circular plates. We calculated the deformation process of square and rectangular thin plates at different mesh scales. The results show that the factors affecting the deformation are related to the Young’s modulus, load, plate length and deformation factor of the material. As for the semi-analytical solution of the deflection function, w(x,y) is found through the Fourier series approximation in the polar coordinate. The consistency of the numerical solution and the theoretical solution is verified. The semi-analytical results are more accurate than the FEM numerical solution, but the derivation process is complicated. In a separate temperature physical field, we established a 2D transient heat conduction model to solve the graphene film. In this paper, three discrete schemes of the transient heat conduction equation of FDM-FEM are given. Finally, this paper chooses the implicit Euler method to be discrete to improve the numerical stability of the time term. Through the comparison of examples, the difference between the adiabatic condition and the convection condition is found by the graphical method and the curve trend. Numerical experiments calculated the case of 0–0.8 s; the results show that near the adiabatic boundary is higher and the graphene is a very good thermal conductivity material.

In order to accurately describe the relationship between temperature and variable force, a 3D dynamic thermal-mechanical coupling model (3D-DTMCM) is proposed. We found that Young’s modulus decreases with increasing temperature, and a model of the laser heating monocrystalline silicon sheet with a periodic motion formula is given. The temperature radiation of the laser heat source can be reflected by the Gaussian distribution characteristics. Under the condition of constant external force load and laser heating for 1 min, the numerical results show that the Young’s modulus decreases with the increase of temperature, but the strain energy density increases slightly with the nonlinear increase of temperature. In addition, the deformation amplitude of the plates in the coupling field is larger than that in the single mechanical field. Finally, a deformation model of the material plate with defects is established, and we also consider comprehensive factors such as high temperature, load, internal mixed cracks, etc. This model will be our future work. In short, our research provides theoretical support for the deformation of different plates and also reflects the value of the coupled model in practical applications. This paper promotes the basic theory and numerical simulation of the mechanics of elastic materials, which also expands the application range of elastic deformation. Our study will provide a new insight into the influence of temperature on the deformation of 2D and 3D materials. Meanwhile, the 3D deformation theory of a multi-physical field model is given, which is advocated for by the physics community and industry currently, which is worth further discussion and study. 

## Figures and Tables

**Table 1:** Classification table of thin plate type and deflection size.

Classification of Plates	Film	Thin Plate	Thick Plate
Range	$\frac{h_{\delta }}{b}<\frac{1}{80}$	$\frac{h_{\delta }}{b}<\frac{1}{5}$	$\frac{h_{\delta }}{b}\geq \frac{1}{5}$
Classification of deflection	Small deflection	Medium deflection	large deflection
Range	$\frac{w}{h_{\delta }}<\frac{1}{5}$	$1<\frac{w}{h_{\delta }}<5$	$\frac{w}{h_{s}}\geq 5$

**Table 2:** Summary of Physical Parameters of Monocrystalline Silicon Materials.

Variable Name	Variable Symbol	Value and Unit
Young’s modulus	*E*	170 Gpa
Poission rate	*v*	0.28
Constant pressure heat capacity	$c_{\rho }$	700 J/(kg·m${}^{3}$)
Thermal conductivity	*k*	130 W/(m·k)
Density	ρ	$2329\;\mathrm{kg}/m^{3}$
Thermal expansion coefficient	α	$2.6\times 10^{-6}\left[1/K\right]$
Refractive index	*n*	3.48
Laser power	$P_{\mathrm{laser}\;}$	10 W
Laser heat flux	$\Phi_{q}$	W/m${}^{2}$
Radiation rate of monocrystalline silicon	$E_{\min}$	0.8

## Data Availability

Data for this article can be accessed publicly.

## Appendix A

This section mainly based on the Kirchhoff assumptions to solve some small deflection problems. It is difficult for the stress on the plate cross-section to meet the actual stress boundary conditions. According to the simplification of Saint-Venant’s theorem, the internal force synthesized by the internal stress components of the plate at the boundary should meet the boundary conditions as much as possible. For the internal force on the yoz cross-section, it is satisfied Formula (A1).

(A1) $$M_{xx}=\int_{-h/2}^{h/2}\sigma_{x}zdz,M_{xy}=\int_{-h/2}^{h/2}\tau_{xy}zdz,Q_{x}=\int_{-h/2}^{h/2}\tau_{xz}dz.$$

In the same way, the internal force of the xoz plane satisfies the relation Formula (A2).

(A2) $$M_{yy}=\int_{-h/2}^{h/2}\sigma_{y}zdz,M_{yx}=\int_{-h/2}^{h/2}\tau_{xy}zdz,Q_{y}=\int_{-h/2}^{h/2}\tau_{yz}dz$$

The relationship between the bending moment M and the strain can be written in the form of a matrix, where $D_{bend}=-Eh^{3}/12\left(1-v^{2}\right)$ is called the flexural rigidity.

(A3) $$\left\{M\right\}=\left(\begin{array}{c} M_{xx}\\ M_{yy}\\ M_{xy} \end{array}\right)=\frac{Eh_{\delta }^{3}}{12\left(1-v^{2}\right)}\left[\begin{array}{ccc} 1 & v & 0\\ v & 1 & 0\\ 0 & 0 & \frac{\left(1-v\right)}{2} \end{array}\right]\left(\begin{array}{c} \chi_{x}\\ \chi_{y}\\ \chi_{xy} \end{array}\right)=\frac{h_{\delta }^{3}}{12}\left[D_{bend}\right]\left\{\chi \right\}$$

Considering that the micro-element body is in the equilibrium state, in which $Q_{x}$ represents the shear force per unit length of the edge dy, the equilibrium equation is established in the *z* direction, and the following expression is established.

(A4) $$-Q_{x}dy-Q_{y}dx-\left(Q_{x}+\frac{\partial Q_{x}}{\partial x}dx\right)dy+\left(Q_{y}+\frac{\partial Q_{y}}{\partial y}dy\right)dx+q\left(x,y\right)dxdy=0$$

After finishing the equation, we can get the Equation (A5).

(A5) $$\frac{\partial Q_{x}}{\partial x}+\frac{\partial Q_{y}}{\partial y}+q\left(x,y\right)=0$$

Then, two component equilibrium equations based on the relationship between Moment and shear stress, we can obtain the Formula (A6).

(A6) $$\frac{\partial M_{xy}}{\partial x}+\frac{\partial M_{yy}}{\partial y}=Q_{y},\;\frac{\partial M_{xy}}{\partial y}+\frac{\partial M_{xx}}{\partial x}=Q_{x}.$$

Then bring Equation (A6) into the Equation (A5), and then, we can get Formula (A7).

(A7) $$\frac{\partial^{2}M_{xx}}{\partial x^{2}}+2\frac{\partial^{2}M_{xy}}{\partial x\partial y}+\frac{\partial^{2}M_{yy}}{\partial y^{2}}+q\left(x,y\right)=0$$

We combine Equations (A7) and (A3), it is easy to get Equation (A8). The deformation Equation (A8) of thin plate is related to material parameters *E* and *v*, which is generally used to solve 2D elastic plate bending problems. Finally, the governing equation of elastic thin plate is abbreviated as Equation (A8).

(A8) $$\frac{\partial^{2}w}{\partial x^{4}}+2\frac{\partial^{4}w}{\partial^{2}x\partial^{2}y}+\frac{\partial^{4}w}{\partial y^{4}}=\frac{q(x,y)}{D_{bend}}$$

Therefore, the governing equation of bending deformation of elastic thin plate is a fourth-order bending equation, which is as follows:

(A9) $$\nabla^{4}w=\frac{q}{D_{bend}}$$

where $D_{bend}=-\frac{Eh^{3}}{12\left(1-v^{2}\right)}$, *E* is the elastic modulus of the material, *h* is the thickness of the plate, *v* is the Possion’s ratio, and q(x,y) is the external force load. Equation (A9) is the governing equation of bending deformation, the specific boundary conditions of Equation (A9), there must be a specific kown function w(x,y).

## Appendix B

The calculation of thin plate deformation is generally based on the change of the internal energy of the material to characterize the size of the deformation, which also can use the plane-wave pseudopotential density functional theory to discuss the thermal expansivity and heat capacity. From the micro perspective of materials’ lattice constants are excellently consistent with the available data of theoretical and experimental studies. In addition, the meshless collocation method can also be used to solve the bending equation of thin plate after energy analysis. Approximate numerical results can also be obtained. If we want to compare the small changes of two different thin plate deformation variables, this paper uses the deformation amplification factor to increase the difference, so that the deformation effect is more obvious and the comparison is convenient. In the following, how to obtain the thin plate bending equation through the energy analysis method is given.

The strain energy generated by the bending of thin plate:

(A10) $$U=\frac{1}{2}{\;\int \int \;}_{V}{\left\{\varepsilon \right\}}^{T}\left\{\sigma \right\}dV$$

The strain energy generated under the action of generalized stress and strain:

(A11) $$U=\frac{1}{2}{\;\int \int \;}_{s}{\left\{\chi \right\}}^{T}\left\{D\right\}\left\{\chi \right\}dxy$$

The external load on the plate is q(x,y) and the potential energy is formed:

(A12) $$\Psi =-W=-{\;\int \int \;}_{S}qwdxy$$

The total potential is:

(A13) $$\begin{array}{cc} \Pi = & U+\Psi =\frac{Eh_{\delta }^{3}}{24\left(1-v^{2}\right)}{\;\int \int \;}_{S}\left\{{\left(\frac{\partial^{2}w}{\partial x^{2}}+\frac{\partial^{2}w}{\partial y^{2}}\right)}^{2}\right.\\ \; & \left.{\;\int \int \;}_{S}-2\left(1-u\right)\left[\frac{\partial^{2}w}{\partial x^{2}}\frac{\partial^{2}w}{\partial y^{2}}-\left(\frac{\partial^{2}w}{\partial x\partial y}\right)\right]\right\}dxdy \end{array}$$

Using the principle of minimum potential energy, the basic differential equation of thin plate bending is obtained, which is also called the biharmonic equation of thin plate. The solution obtained by FEM is also convergent.

(A14) $$\frac{Eh_{\delta }^{3}}{12\left(1-v^{2}\right)}\left(\frac{\partial^{4}w}{\partial x^{4}}+2\frac{\partial^{4}w}{\partial x^{2}\partial y^{2}}+\frac{\partial^{4}w}{\partial y^{4}}\right)=q\left(x,y\right)$$

## Appendix C

(1) Simple support boundary conditions.

If the edge x=a of the rectangle plate is a simply supported edge, then the deflection at this place is w(x,a)=w(a,y)=0, at the same time the boundary here can be freely rotated, and the simply supported edge has no bending moment $M_{xx}$, namely.

(A15) $${\left.M_{xx}\right|}_{x=a}=-D_{bend}\left(\frac{\partial^{2}w}{\partial x^{2}}+v\frac{\partial^{2}w}{\partial y^{2}}\right)=0$$

Since *w* = 0, it indicates that when and only when $\frac{\partial^{2}w}{\partial x^{2}}=\frac{\partial^{2}w}{\partial y^{2}}=0$, the boundary condition of simply supported edge is

(A16) $${\left.w\right|}_{k=a}=0,\;\frac{\partial^{2}w}{\partial x^{2}}=0$$

(2) The rolling boundary condition:

(A17) $$w_{x}\left(a,t\right)=w_{xxx}\left(a,t\right)=0,\;a=0\;\;\mathrm{or}\;L=0$$

## Appendix D. Theory of Circular Plate Bending

Considering the deformation of the circular plate under normal load, the polar coordinate (r,θ) can be used for modeling, we convert Cartesian coordinate system to polar coordinate system. *r* is the distance from the center of the circular plate, and θ is the polar angle of counterclockwise rotation. Using the standard coordinate transformation formula, the relationship between the first-order partial derivative of function *f* in polar and rectangular coordinate systems is derived as follows:

(A18) $$\frac{\partial w}{\partial x}=\cos\theta \frac{\partial w}{\partial r}-\frac{\sin\theta }{r}\frac{\partial w}{\partial \theta }$$

(A19) $$\frac{\partial w}{\partial y}=\sin\theta \frac{\partial w}{\partial r}+\frac{\cos\theta }{r}\frac{\partial w}{\partial \theta }$$

According to the semi-analytical Fourier series method, the series approximation term Equation A21 is introduced into the fourth-order deflection differential Equation A20, and then the coefficients of the corresponding Fourier approximation term are solved. Finally, we can use the Fourier series term to approximate the deflection function w(r,θ). The fourth-order deflection differential equation is shown as:

(A20) $$\nabla^{4}w=-\frac{w}{D_{bend}}=-\frac{12\left(1-v^{2}\right)w}{Eh^{3}}$$

The general solution to the lateral displacement of the plate bending equation can be approximated using the Fourier series.

(A21) $$w\left(r,\theta \right)=\frac{1}{2}p_{0}\left(r\right)+\sum_{n=1}^{\infty }p_{n}\left(r\right)\cos n\theta +q_{n}\left(r\right)\sin n\theta$$

where $p_{n}\left(r\right)$ and $q_{n}\left(r\right)$ are unknown functions, and the complex representation of the deflection function w(r,θ) is

(A22) $$w\left(r,\theta \right)=\sum_{n=1}^{\infty }F_{n}\left(r\right)\exp\left(-in\theta \right)$$

In Formula (A22), *i* is an imaginary unit, and Euler’s formula for complex exponents is used:

(A23) exp(−inθ)=cos(nθ)−isin(nθ)

To facilitate calculation, we introduce complex function $F_{n}\left(r\right)$ when n≥0, we can get

(A24) $$F_{n}\left(r\right)=\frac{1}{2}\left[p_{n}\left(r\right)+iq_{n}\left(r\right)\right]$$

Similarly, when the complex function $F_{n}\left(r\right)$ is n<0, we can get the Formula (A25).

(A25) $$F_{n}\left(r\right)=\frac{1}{2}\left[p_{n}\left(r\right)-iq_{n}\left(r\right)\right]$$

In addition, $F_{n}\left(r\right)=F_{-n}^{*}\left(r\right)$ can be obtained from the complex conjugate relationship, and then the Fourier series approximation term is introduced into the control equation of thin plate bending. We can get Equation (A26) by substituting $w\left(r,\theta \right)=\sum_{n=1}^{\infty }F_{n}\left(r\right)\exp\left(-in\theta \right)$ approximation into harmonic differential operator.

(A26) $$\nabla^{4}w\left(r,\theta \right)=\sum_{n=-\infty }^{\infty }\varphi_{n}\exp\left(-in\theta \right)=-\frac{w}{D_{bend}}$$

In the Equation (A26), $\varphi_{n}$ is the coefficient corresponding to Fourier series.

(A27) $$\varphi_{n}=F_{n}^{\left(4\right)}+\frac{2}{r}F_{n}^{\left(3\right)}-\frac{1+2n^{2}}{r^{2}}F_{n}^{\left(2\right)}+\frac{1+2n^{2}}{r^{3}}F_{n}^{\prime }+\frac{n^{2}\left(n^{2}-4\right)}{r^{2}}F_{n}$$

The analytical solution of non-homogeneous differential equation is generally composed of special solution and homogeneous general solution. The special solution contains the right-hand term function, and the second general solution corresponds to the case of $w=0,\;\varphi_{n}=0$. The Fourier coefficients of the homogeneous solution are shown below.

(A28) $$F_{0}\left(r\right)=A_{0}r^{2}+B_{0}r^{2}\ln\frac{r}{a}+C_{0}\ln\frac{r}{a}+D_{0}$$

(A29) $$F_{1}\left(r\right)=A_{0}r^{3}+B_{1}\ln\frac{r}{a}+C_{1}r+D_{1}\frac{1}{r}$$

(A30) $$F_{n}\left(r\right)=A_{n}r^{n+2}+B_{n}r^{n}+C_{n}r^{-n+2}+D_{n}r^{-n}$$

Among them, *a* is the radius of the plate, constant $A_{i},B_{i},C_{i}$ and $D_{i}$ determined by boundary conditions, when the load density function *f* is uniform on the whole circular plate, there is a suitable special solution:

(A31) $$w\left(r,\theta \right)=-\frac{f(r,\theta )}{64D_{bend}}r^{4}=-\frac{3\left(1-v^{2}\right)f\left(r,\theta \right)}{16Eh^{3}}r^{4}$$

The corresponding second-order homogeneous general solution is:

(A32) $$w\left(r,\theta \right)=-\frac{f(r,\theta )}{64D_{bend}}{\left(a-r\right)}^{2}=-\frac{3\left(1-v^{2}\right)f\left(r,\theta \right)}{16Eh^{3}}{\left(a-r\right)}^{2}$$
